# Supplementary material for: Compromised Metabolic Reprogramming Is an Early Indicator of CD8+ T Cell Dysfunction during Chronic Mycobacterium tuberculosis Infection
Source: Cell Rep. 2019 Dec 10;29(11):3564–3579.e5. doi: 10.1016/j.celrep.2019.11.034 (PMC6915325; doi:10.1016/j.celrep.2019.11.034)
Supplement: Document S1. Figures S1–S6 [file mmc1.pdf]

Cell Reports, Volume *29*

Supplemental Information

Compromised Metabolic Reprogramming Is an Early Indicator of CD8$^+$ T Cell Dysfunction during Chronic *Mycobacterium tuberculosis* Infection

Shannon L. Russell, Dirk A. Lamprecht, Tawanda Mandizvo, Terrence T. Jones, Vanessa Naidoo, Kelvin W. Addicott, Chivonne Moodley, Bongani Ngcobo, David K. Crossman, Gordon Wells, and Adrie J.C. Steyn

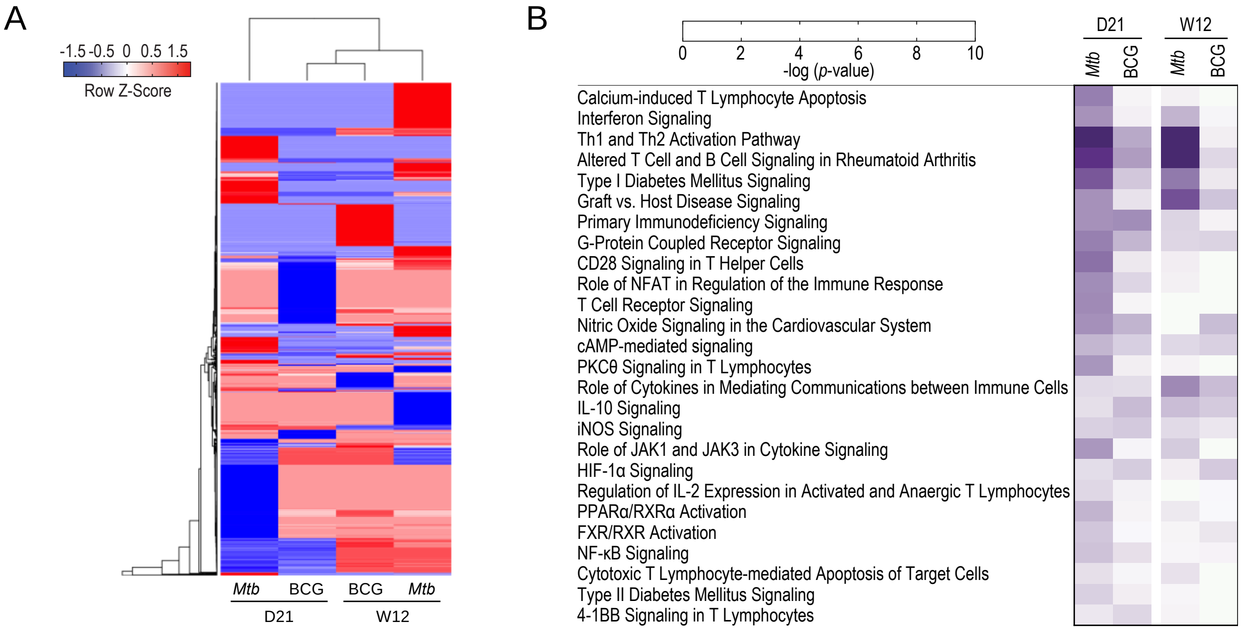

Figure S1. Variation in global gene expression patterns and enriched pathway analysis in lung CD8$^+$ T cells during *Mtb* or BCG infection. (Related to Figure 1.) RNA was extracted from purified CD8$^+$ T cells from the lungs of *Mtb* or BCG-infected mice at D21 and W12, sequenced and analyzed for differences in global gene expression patterns. **(A)** Global differential gene expression patterns of CD8$^+$ T cells from *Mtb* and BCG-infected animals at D21 and W12 (relative to UI). Clustering indicates the relatedness between samples as defined by heatmap.2 in R. **(B)** The top canonical pathways are shown based on enrichment score (Fisher's exact test *P*-value), defined by QIAGEN's Ingenuity® Pathway Analysis software. All comparisons are relative to expression levels of CD8$^+$ T cells purified from the lungs of UI mice.

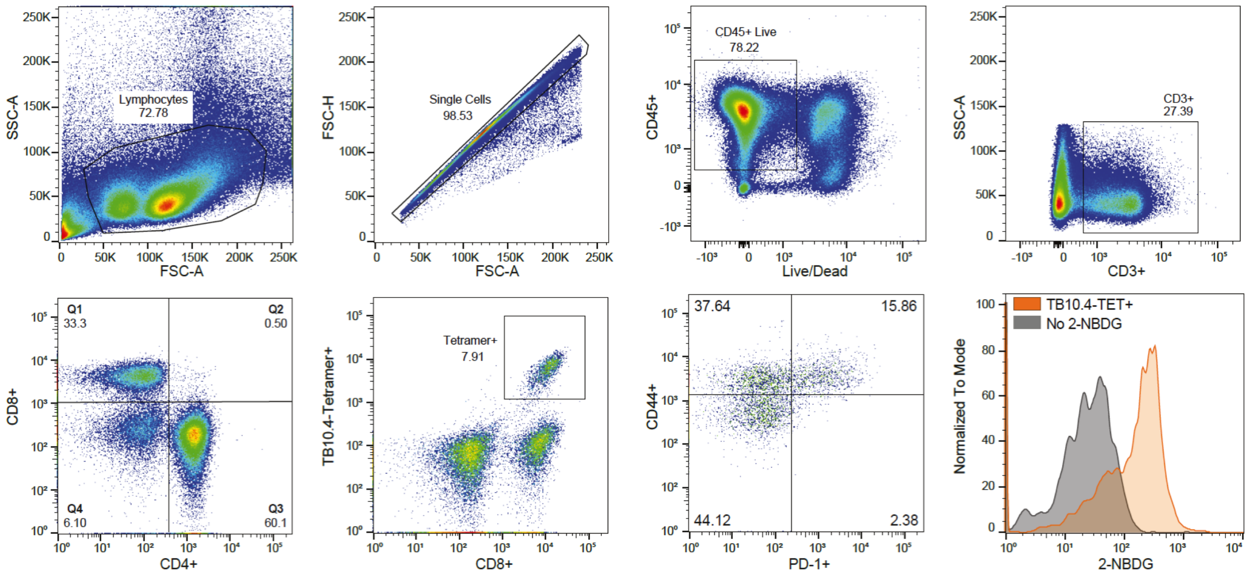

Figure S2. Gating strategy for flow cytometry analysis of CD8^{+} T cells. (Related to Figure 2.)

Purified single cell suspensions prepared from the lungs of uninfected or infected mice were stained with fluorochrome-conjugated antibodies and analyzed on the BD Fortessa or FACS Aria II. Using FlowJo software (TreeStar, V10.1), total lymphocytes and singlets were gated using FSC and SSC parameters, followed by live, CD45^{+} cells. Cytotoxic T lymphocytes were identified by gating on the CD3^{+} population, followed by those that stained CD8^{+}CD4^{-}. *Mtb*-specific CD8^{+} T cells were identified as those that stained TB10.4 tetramer^{+} within the CD3^{+}CD8^{+}CD4^{-} subset. Cells were further gated on CD44^{+} expression to identify T effectors, and subsequently PD-1 or CTLA-4 (not shown) to examine co-inhibitory receptor expression. The cell population of interest was examined for 2-NBDG uptake (measured by mean fluorescence intensity, or MFI) if glucose uptake was assayed; this was always normalized to the MFI of unstained cells.

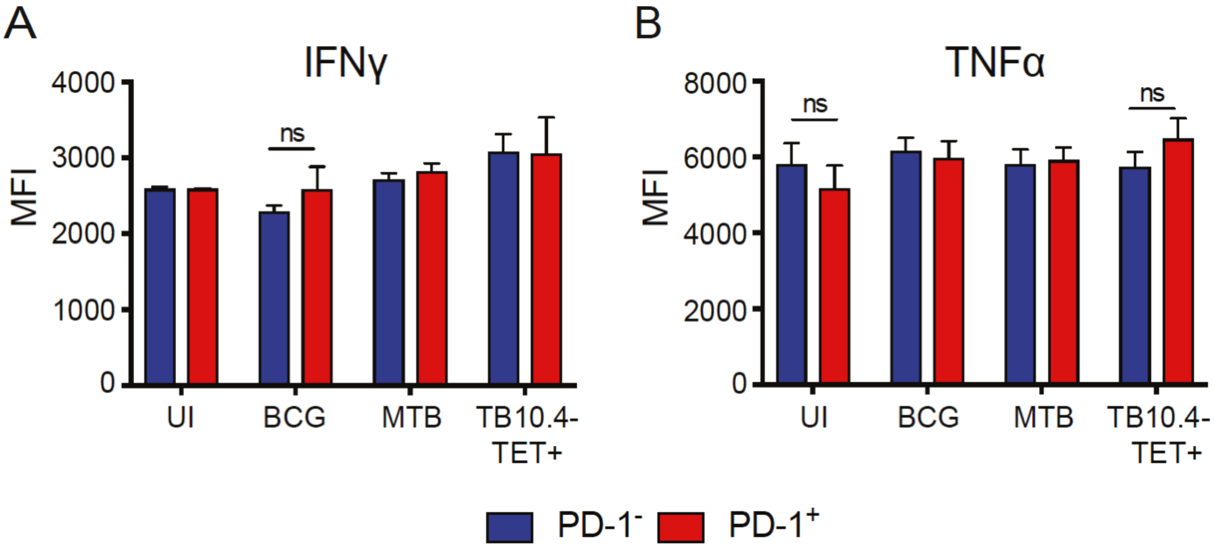

Figure S3. Cytokine production in CD8$^+$ T cells expressing PD-1 receptor. (Related to Figure 2.)

Purified single cell suspensions prepared from the lungs of uninfected or infected mice were stimulated with PMA/ionomycin for 4 hours and then stained with fluorochrome-conjugated antibodies. CD8$^+$ T cells were gated based on their expression of PD-1 receptor, and intracellular cytokine production was measured based on the mean fluorescence intensity of the antibody used to detect the cytokine of interest. Data shown is from D35 post-infection, although reflects similar trends at D21 and W12. Data are representative of three independent experiments (5 mice per experimental group). ns, not significant, by unpaired Student's t-test. Error bars are mean ± SD.

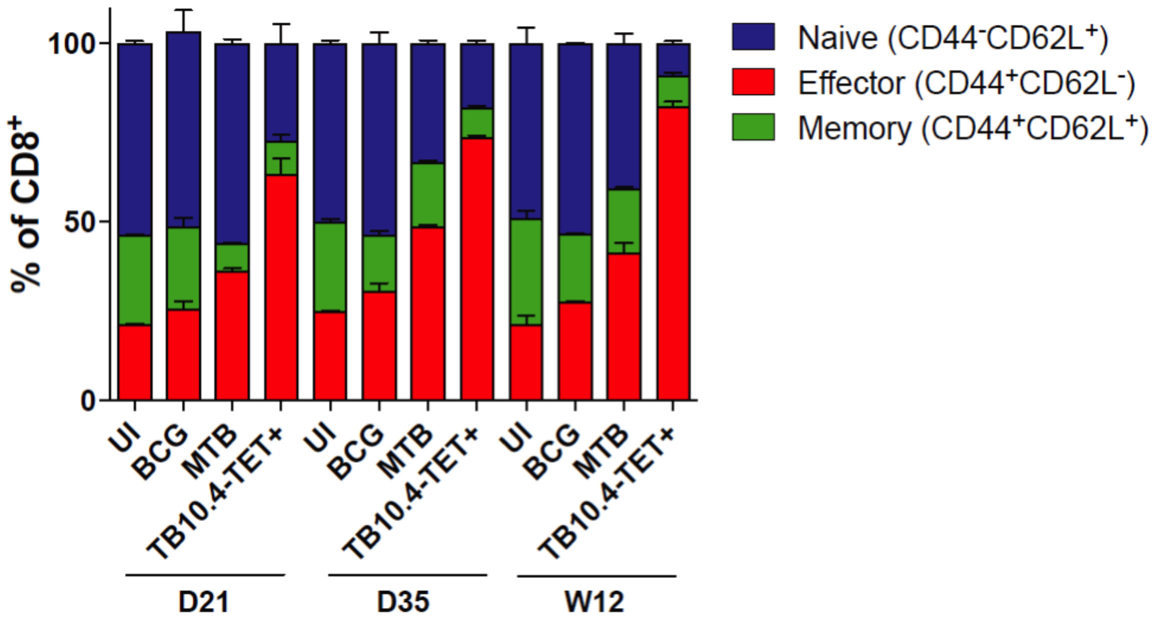

Figure S4. Naïve, effector and memory CD8$^+$ T cell populations over time in UI, BCG and *Mtb*-infected mice. (Related to Figure 3.) Expression of CD44 and CD62L were used to estimate the number of naïve (CD44$^-$CD62L$^+$), effector (CD44$^+$CD62L$^-$) and memory (CD44$^+$CD62L$^+$) CD8$^+$ T cells present in purified single cell suspensions prepared from the lungs of uninfected or infected mice on D21, D35 and W12 post-infection using unfixed flow cytometry. Data are representative of two independent experiments (5 mice per experimental group). Error bars are mean ± SD.

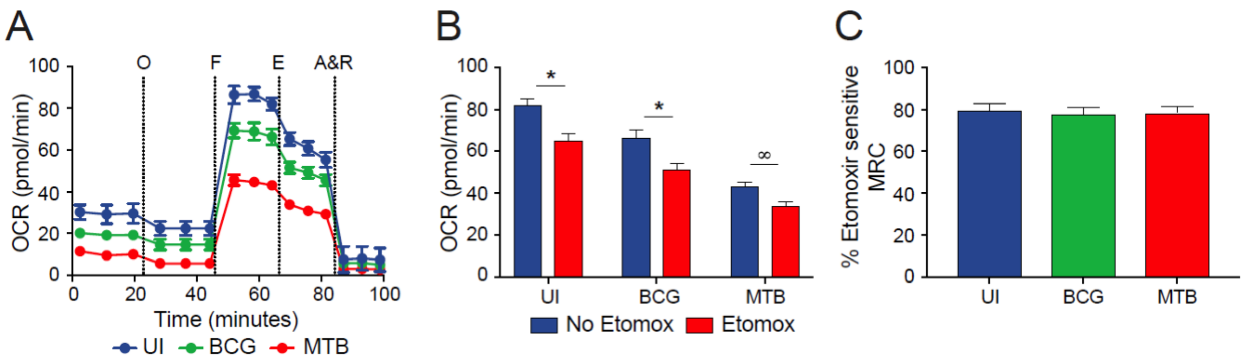

Figure S5. Fatty acid oxidation contributes equally to OCR in CD8⁺ T cells, regardless of infection status. (Related to Figure 3.) Extracellular flux (XF) analysis was performed on purified lung CD8⁺ T cells during chronic *Mtb* or BCG infection. **(A)** OCR measured during a cell mito stress test (CMST), performed with an extra infection of the Cpt1a inhibitor, etomoxir (E) after uncoupling with FCCP. **(B)** OCR before and after etomoxir injection (inhibits fatty acid oxidation, FAO) and **(C)** % etomoxir-sensitive OCR (OCR not due to FAO). Data shown is from D35 post-infection, although reflects similar trends at D21 and W12. UI, uninfected; OCR, oxygen consumption rate; ECAR, extracellular acidification rate; O, oligomycin; F, FCCP or carbonyl cyanide p-trifluoromethoxyphenylhydrazone; E, etomoxir; A&R, antimycin A and rotenone. Data are representative of two independent experiments (5 mice per experimental group). ∞ $p \leq 0.005$; * $p \leq 0.001$, by unpaired Student's t-test or one-way ANOVA. Error bars are mean ± SD.

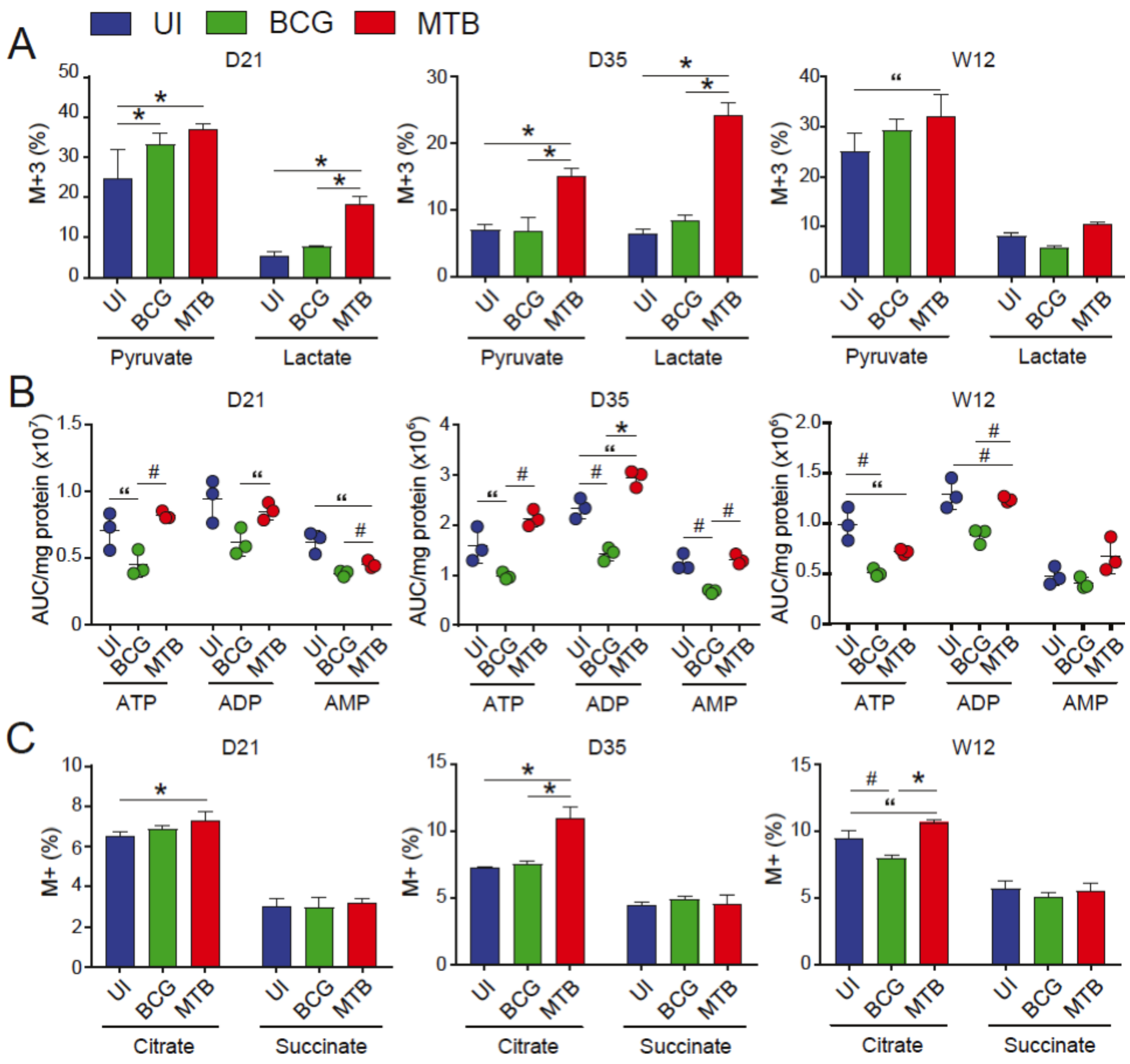

Figure S6. *Mtb* infection increases metabolic flux through glycolysis to generate ATP via substrate-level phosphorylation. (Related to Figure 3.) Metabolite pools were generated in CD8$^+$ T cells purified from UI, *Mtb* or BCG-infected animals at D21, D35 or W12 post-infection following incubation for 1h at 37 °C in ^{13}C-glucose-containing media. **(A)** Pyruvate and lactate M+3 isotopologues, **(B)** absolute ATP, ADP and AMP levels and **(C)** citrate and succinate combined M+ isotopologues detected in metabolite extracts, as quantified by LC-MS. UI, uninfected. Stats unless otherwise indicated, are relative to UI. Data are representative of two independent experiments (N = 5 mice per group). “ $p \leq 0.05$; # $p \leq 0.01$; * $p \leq 0.001$, by one-way ANOVA. Error bars are mean ± SD.
