# Supplementary material for: Compromised Metabolic Reprogramming Is an Early Indicator of CD8+ T Cell Dysfunction during Chronic Mycobacterium tuberculosis Infection
Source: Cell Rep. 2019 Dec 10;29(11):3564–3579.e5. doi: 10.1016/j.celrep.2019.11.034 (PMC6915325; doi:10.1016/j.celrep.2019.11.034)
Supplement: Document S2. Article plus Supplemental Information [file mmc2.pdf]

Cell Reports

Article

Compromised Metabolic Reprogramming Is an Early Indicator of CD8$^+$ T Cell Dysfunction during Chronic *Mycobacterium tuberculosis* Infection

Graphical Abstract

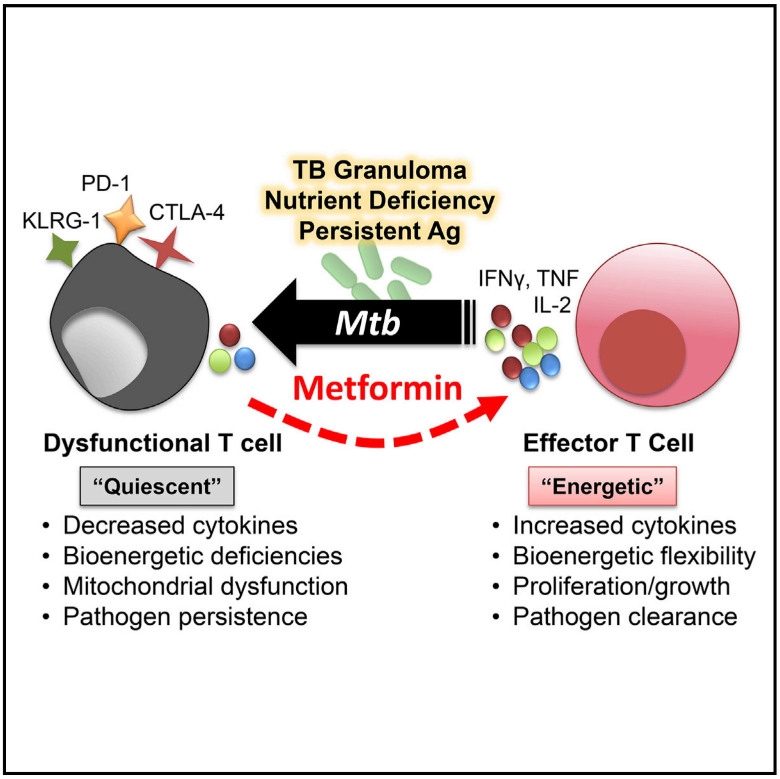

Highlights

- T cells from *Mtb* and BCG infections have unique metabolic and functional signatures
- Mitochondrial metabolism deteriorates in effector T cells as *Mtb* infection persists
- Metformin rejuvenates mitochondrial metabolism in T cells from *Mtb*-infected mice
- The breakdown of *Mtb* immunity during chronic disease is linked to immunometabolism

Authors

Shannon L. Russell, Dirk A. Lamprecht, Tawanda Mandizvo, ..., David K. Crossman, Gordon Wells, Adrie J.C. Steyn

Correspondence

asteyn@uab.edu

In Brief

T cell dysfunction contributes to the development of many chronic infections and cancer. Russell et al. show that chronic *Mtb* infection leads to a progressive decline in the metabolic health of effector T cells that reside in lung lesions. This metabolic reprograming can be reversed by treatment with metformin.

Russell et al., 2019, Cell Reports *29*, 3564–3579
December 10, 2019 © 2019 The Author(s).
https://doi.org/10.1016/j.celrep.2019.11.034

CellPress

OPEN ACCESS
CellPress

Cell Reports
Article

Compromised Metabolic Reprogramming Is an Early Indicator of CD8$^+$ T Cell Dysfunction during Chronic *Mycobacterium tuberculosis* Infection

Shannon L. Russell,[1,7] Dirk A. Lamprecht,[1,8] Tawanda Mandizvo,[1] Terrence T. Jones,[2] Vanessa Naidoo,[1] Kelvin W. Addicott,[1] Chivonne Moodley,[1,9] Bongani Ngcobo,[1] David K. Crossman,[3] Gordon Wells,[1] and Adrie J.C. Steyn[1,4,5,6,10,*]
[1]Africa Health Research Institute, Durban 4001, South Africa
[2]Health Science Center (UTHSC), Department of Medicine, University of Tennessee, Memphis, TN 38163, USA
[3]Heflin Center for Genomic Science, Department of Genetics, University of Alabama, Birmingham, AL 35487, USA
[4]Department of Microbiology, University of Alabama, Birmingham, AL 35487, USA
[5]Center for AIDS Research (CFAR), University of Alabama, Birmingham, AL 35487, USA
[6]Center for Free Radical Biology (CFRB), University of Alabama, Birmingham, AL 35487, USA
[7]Present address: Public Health Laboratory, British Columbia Center for Disease Control, Vancouver, BC V5Z 4R4, Canada
[8]Present address: Janssen Pharmaceutica, Turnhoutseweg 30, 2340 Beerse, Belgium
[9]Present address: Department of Microbiology and Immunology, Tulane University, New Orleans, LA 70112, USA
[10]Lead Contact
*Correspondence: asteyn@uab.edu
https://doi.org/10.1016/j.celrep.2019.11.034

SUMMARY

The immunometabolic mechanisms underlying suboptimal T cell immunity in tuberculosis remain undefined. Here, we examine how chronic *Mycobacterium tuberculosis* (*Mtb*) and *M. bovis* BCG infections rewire metabolic circuits and alter effector functions in lung CD8$^+$ T cells. As *Mtb* infection progresses, mitochondrial metabolism deteriorates in CD8$^+$ T cells, resulting in an increased dependency on glycolysis that potentiates inflammatory cytokine production. Over time, these cells develop bioenergetic deficiencies that reflect metabolic "quiescence." This bioenergetic signature coincides with increased mitochondrial dysfunction and inhibitory receptor expression and was not observed in BCG infection. Remarkably, the *Mtb*-triggered decline in T cell bioenergetics can be reinvigorated by metformin, giving rise to an *Mtb*-specific CD8$^+$ T cell population with improved metabolism. These findings provide insights into *Mtb* pathogenesis whereby glycolytic reprogramming and compromised mitochondrial function contribute to the breakdown of CD8$^+$ T cell immunity during chronic disease, highlighting opportunities to reinvigorate immunity with metabolically targeted pharmacologic agents.

INTRODUCTION

Our current understanding of what governs tuberculosis (TB) disease progression and control in humans is limited by the lack of existing knowledge about how protective immune responses are generated within TB lesions. The central dogma suggests that within TB lesions, infected macrophages are activated by antigen-specific CD4$^+$ T cells that secrete interferon-gamma (IFN-γ), restricting the growth and dissemination of *Mycobacterium tuberculosis* (*Mtb*) (Nunes-Alves et al., 2014). This dogma has since been revised to recognize the importance of additional T cell subsets that elicit protective immune responses to *Mtb*. There is increasing evidence that CD8$^+$ T cells are important for effective control of *Mtb* because they kill infected host cells directly and facilitate long-lived immunological memory (Chen et al., 2009; Flynn et al., 1992; Stenger et al., 1998; van Pinxteren et al., 2000). Humans fail to generate robust CD8$^+$ T cell memory during *Mtb* infection, even after successful treatment (Verver et al., 2005); similar findings have been observed in animal models (Carpenter et al., 2016; Einarsdottir et al., 2009). Poor memory T cell responses also remain a caveat of most existing TB vaccine candidates to date (Fine, 1995; Orme, 1999) and were thought to have contributed to the failure of the highly anticipated MVA85A vaccine trial (Tameris et al., 2013). Failure to develop and sustain this essential antigen-experienced CD8$^+$ T cell population during *Mtb* infection suggests that there may be a defect in key regulatory mechanisms that foster the differentiation of CD8$^+$ effector T cells into long-lived, multi-potent memory cells.

T cell dysfunction plays a key role in the loss of immune control and aberrant inflammation associated with some chronic viral infections and cancers. There is evidence from chronic viral infections such as lymphocytic choriomeningitis virus (LCMV) and hepatitis B virus (HBV) that persistent antigen exposure compromises CD8$^+$ T cell function, driving the cell into a state of exhaustion marked by an altered global transcriptional program, metabolic insufficiencies, increased expression of inhibitory markers (PD-1, CTLA-4, LAG-3, and 2B3), and poor effector function (Bengsch et al., 2016; Blackburn et al., 2009; Schurich et al., 2016; Wherry et al., 2007). This phenomenon is also observed in the nutrient-deficient tumor microenvironment, where tumor-infiltrating CD8$^+$ T

 © 2019 The Author(s).
This is an open access article under the CC BY license (http://creativecommons.org/licenses/by/4.0/).

lymphocytes (TILs) fail to elicit productive anti-tumor responses (Crespo et al., 2013). The availability of nutrients (or lack thereof) within densely packed TB lesions could have similar detrimental effects on T cell responses during chronic *Mtb* infection. Increased expression of inhibitory markers, as well as the terminal differentiation marker CD57 (KLRG-1), have been detected on antigen-specific T cells from human TB patients (Lee et al., 2015; Singh et al., 2017; Wang et al., 2011). This work, in conjunction with functional studies in mice (Jayaraman et al., 2016), suggests that $CD8^+$ T cell immunity is suboptimal during chronic *Mtb* infection because of T cell exhaustion.

Distinct metabolic programs are initiated upon T cell activation, differentiation, and effector and memory transitions in the lymphocyte life cycle (Buck et al., 2015). This metabolic reprogramming can be altered by chemical signals from the surrounding environment or immune checkpoint regulators (e.g., PD-1, CTLA-4) on the cell surface, limiting effector T cell differentiation and function (Patsoukis et al., 2015). For instance, functional impairments in $CD8^+$ T cells in the tumor microenvironment have been linked to upstream metabolic dysregulation (Ho et al., 2015; Siska et al., 2017). Because many parallels exist between the tumor microenvironment and TB lesions, similar mechanisms could be responsible for the breakdown in T cell-mediated immunity observed during chronic *Mtb* infection.

Increased TB risk is associated with several immunometabolic disease states, including type 2 diabetes and malnutrition (Dooley and Chaisson, 2009; Jeon and Murray, 2008; Lönnroth et al., 2010), suggesting that an important component of TB etiology involves immunometabolic derangement. Despite decades of extensive immunological characterization of the immune response during *Mtb* infection, little is known about how metabolic reprogramming contributes to the development of dysfunctional immune responses in TB. Recent work from our lab has revealed that *Mtb* rewires macrophage energy metabolism to support its survival in the host by decelerating flux through glycolysis and the tricarboxylic acid (TCA) cycle and limiting ATP availability (Cumming et al., 2018). Further characterization of these events *in vivo* is required to understand how metabolic reprogramming of specific immune cell populations (i.e., macrophages, neutrophils, T cells, etc.) contributes to *Mtb* persistence within TB lesions.

In this study, we hypothesize that *Mtb* maintains persistence during chronic infection by inducing environmental cues that lead to the metabolic and functional deterioration of $CD8^+$ T cell responses. To test this hypothesis, we used a combination of flow cytometry, bioenergetic, metabolomic, and transcriptomic analyses to compare $CD8^+$ T cell populations purified from mice infected with virulent *Mtb*, or the non-pathogenic vaccine strain, *Mycobacterium bovis* Bacillus Calmette-Guérin (BCG). Comparing the effects of these two distinct mycobacterial infections on $CD8^+$ T cell immunometabolism over time allows us to identify signatures that correspond with *Mtb* pathogenesis. In doing so, we have identified important interactions between metabolism and the breakdown of *Mtb* immunity that lay the groundwork for future mechanistic studies in this interdisciplinary field.

RESULTS

To test our hypothesis that chronic *Mtb* infection coincides with metabolic alterations in the $CD8^+$ T cell population that impede downstream effector function and hinder disease resolution, we compared mice infected with pathogenic *Mtb* with mice infected with the non-pathogenic vaccine strain, *M. bovis* BCG. We compared the metabolic and functional responses of total and *Mtb*-specific $CD8^+$ T cells from *Mtb*-infected mice with those from uninfected (UI) and BCG-infected mice at early (day 21 [D21], D35) and late (week 12 [W12]) time points post-infection (Figure 1A). Using this experimental approach, we investigated how the metabolic and immunologic characteristics of $CD8^+$ T cells coincided with two kinetically distinct bacterial infections in the lung over time (Figure 1B).

$CD8^+$ T Cell Transcriptional Signatures Diverge between *Mtb* and BCG Infections

To examine the molecular signatures present in $CD8^+$ T cells over the course of infection with pathogenic or non-pathogenic mycobacteria, we purified total $CD8^+$ T cells from the lungs of mice infected with *Mtb* or BCG at D21 (early) and W12 (late) post-infection and compared them with cells from UI mice. Transcriptional profiling revealed 2,990 and 1,766 genes were differentially regulated in $CD8^+$ T cells from *Mtb*-infected mice compared with BCG at D21 and W12, respectively. Compared with UI mice, 429 differentially expressed genes were common to both infections at D21, while at W12, this number had decreased to 341 (Figure 1C). The magnitude of genes differentially expressed during *Mtb* infection relative to BCG when normalized to UI (1,747 versus 703 at D21 and 1,567 versus 821 at W12 for *Mtb* and BCG, respectively; Figure 1C), as well as the influence of time (D21 versus W12), suggests that different infection kinetics early on could play a role in promoting protective versus pathogenic T cell responses over time. Principal-coordinate analysis (Figure 1D) revealed greater similarities in global gene expression patterns between UI and BCG-infected mice compared with *Mtb*, suggesting that *Mtb* infection more substantially alters T cell function. A heatmap and hierarchical clustering of these differentially expressed genes (relative to UI) further illustrates that the gene expression patterns are more similar at D21 and W12 in BCG infection than *Mtb* at the same time points (Figure S1A). Next, we used Ingenuity Pathway Analysis (IPA) to identify canonical pathways significantly activated/repressed (*Z* score; Figure 1E) or enriched (p value; Figure S1B) in these $CD8^+$ T cell populations. Most pathways affected by infection—especially those pertaining to T cell activation, effector functions, apoptosis, and metabolism—became activated during *Mtb* infection compared with their UI counterparts, particularly at W12; in contrast, these pathways were not activated in T cells from BCG-infected mice at D21, only at W12, further emphasizing the differences in T cell activation programs induced by these two infections (Figure 1E). Pathways linked to immunometabolism were of particular interest, including type 1 and type 2 diabetes mellitus signaling, PPARα/RXRα activation, cyclic AMP (cAMP)-mediated signaling, and HIF1α signaling. Analysis of selected differentially expressed genes revealed significant upregulation of

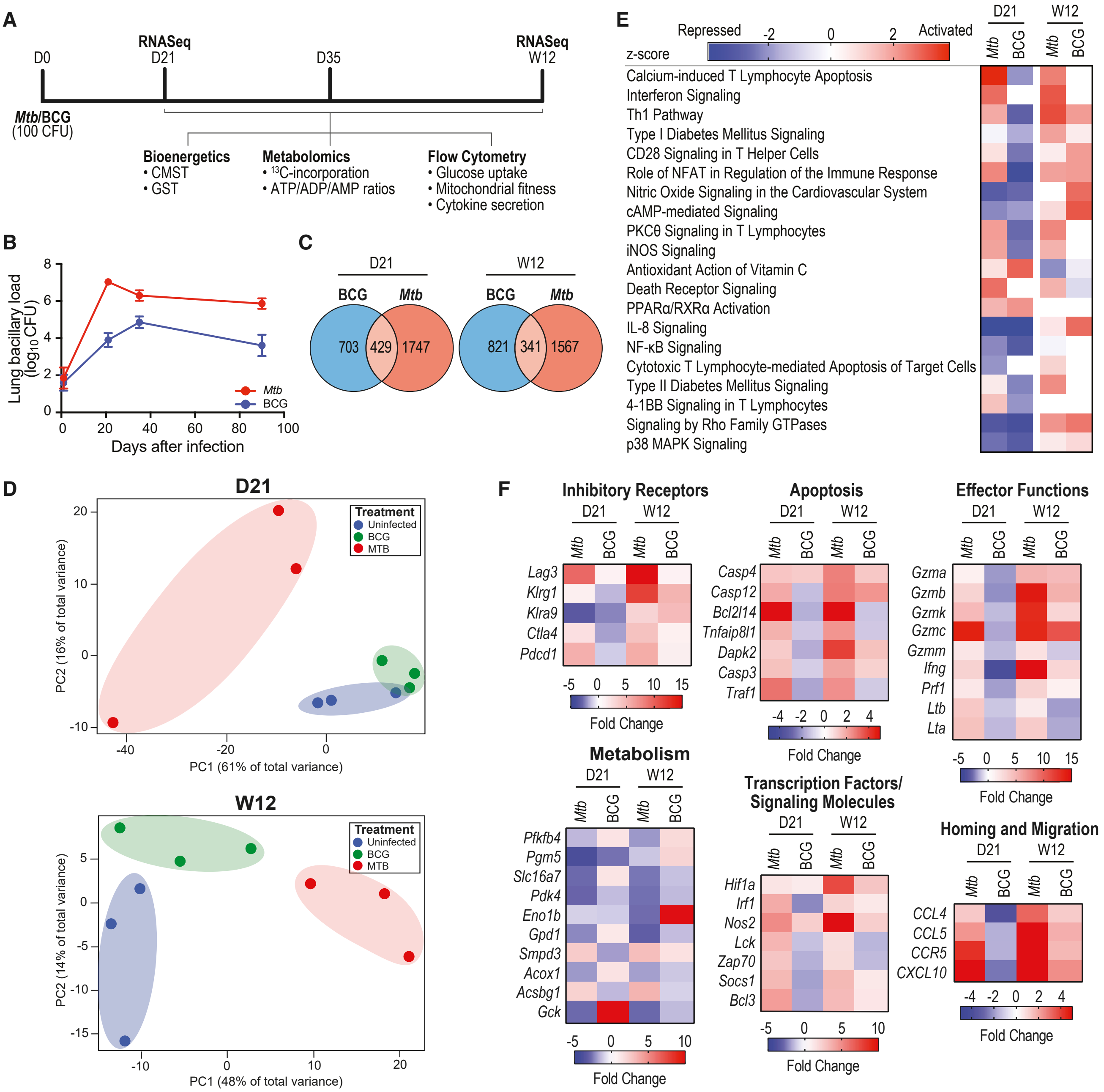

Figure 1. Divergence in CD8^{+} T Cell Transcriptional Signatures Early and Late in *Mtb* Infection Compared with BCG

(A) Experimental timeline.

(B) Colony-forming units (CFUs) in the lung over the course of infection.

(C) The number of genes differentially expressed by CD8^{+} T cells purified from the lungs of infected mice at D21 and W12 relative to uninfected (UI) mice.

(D) Principal-component analysis (PCA) of gene expression patterns from individual mice at D21 and W12.

(E) *Z* scores of differentially expressed genes (fold change $\geq \pm 2$, $p < 0.05$) grouped according to canonical pathways identified by Ingenuity Pathway Analysis (QIAGEN). Red, activated; blue, repressed; white, not significantly altered. Only selected pathways are shown.

(F) Select differentially expressed genes grouped according to functional category.

Heatmap shading is based on fold-change gene expression values relative to UI mice at the designated time point. Genes considered significant have fold-change values $\geq \pm 2$. Genes not assigned fold-change values (undetected) are shown in white. CFU data are representative of three independent experiments (n = 5 mice per group). Error bars are mean ± SD.

genes pertaining to effector function, such as granzymes, perforins, and lymphotoxins, and genes relating to homing and T cell migration, as well as striking increases in genes associated with inhibitory receptor expression and apoptosis; these trends were most prominent in T cells from *Mtb*-infected mice at W12 (Figure 1F). Several genes involved in metabolism were also differentially expressed, suggesting that $CD8^+$ T cells from *Mtb* and BCG infections have distinct metabolic and functional signatures (Figure 1F). Interestingly, most of the metabolic genes we detected decreased in expression relative to UI and BCG-infected mice, except for *Smpd3* and *Acsbg1*, genes involved in lipid metabolism.

These data highlight differences in the molecular signatures identified in lung $CD8^+$ T cell populations over time in *Mtb*- and BCG-infected mice. $CD8^+$ T cells from *Mtb* infection are highly activated at D21; expression of genes associated with this inflammatory transcriptomic signature continues to increase at W12, coinciding with the upregulation of genes associated with T cell exhaustion (Wherry et al., 2007), suggesting that prolonged activation and inflammatory signaling may impair T cell function over time. In contrast, BCG infection induces a transcriptomic signature that appears to repress inflammation early on, suggesting that the $CD8^+$ T cell response may be more fine-tuned to control inflammation and resolve infection. These data provide fresh insight into how metabolism and effector functions are closely linked and identify key nodes of regulation that could be targeted to prevent the immunopathology associated with chronic TB disease.

Inhibitory Receptor Expression Is Increased upon *Mtb* Infection, Corresponding with Decreased Glucose Uptake

Transcriptional profiling of $CD8^+$ T cells from *Mtb*-infected mice revealed that by W12, inhibitory receptor expression (*Pdcd1*, *Ctla4*, *Lag3*) was significantly upregulated relative to cells from UI and BCG-infected mice. Upregulation of these receptors could be indicative of functional exhaustion or restrained T cell activation, a mechanism intended to limit lethal immune-mediated pathology (Sakai et al., 2016). We observed an increase in the proportion of PD-1^+ $CD8^+$ T cells in *Mtb*-infected mice at early and late time points post-infection compared with BCG (Figure 2A). An even higher percentage of $CD8^+$ T cells were CTLA-4^+ (Figure 2B). These markers were further increased on *Mtb*-specific T cells, identified using the major histocompatibility complex (MHC) I-restricted $TB10.4_{4-11}$ tetramer (TB10.4-TET^+ cells). Co-expression of multiple inhibitory receptors on the cell surface has been suggested to be an early indicator of declining effector function (Wherry and Kurachi, 2015). Although most CTLA-4^+ cells did not co-express PD-1, CTLA-4^+PD-1^+ co-expression was increased on TB10.4-TET^+ cells by W12 (Figure 2C).

Transcriptomic analyses also indicated upregulation of *Klrg1* in $CD8^+$ T cells from *Mtb*-infected mice at W12 post-infection. We observed a steady increase in the proportion of KLRG-1^+ cells over time in total and *Mtb*-specific $CD8^+$ T cells from *Mtb*-infected mice, and by W12, KLRG-1 was expressed on nearly 80% of TB10.4-TET^+ cells (Figure 2D).

Next, we tested whether the transcriptional changes we observed in metabolic genes could be linked to differences in glucose uptake. Glucose uptake was diminished in $CD8^+$ T cells from *Mtb*-infected mice at D21, D35, and W12 post-infection (Figures 2E and 2F). This effect was enhanced in TB10.4-TET^+ cells. On the basis of recent evidence that PD-1 alters metabolic reprogramming in T cells and hinders the transition from oxidative phosphorylation (OXPHOS) to glycolysis during effector differentiation (Patsoukis et al., 2015), we looked at expression of PD-1 in the context of glucose uptake. To test whether there was a reciprocal relationship between glucose uptake and PD-1 expression, we gated on PD-1^+ and PD-1^- populations in activated $CD44^+CD8^+$ T cells (gating strategy, Figure S2). Glucose uptake was reduced in PD-1^+ cells compared to PD-1^- cells, except in TB10.4-TET^+ cells, in which glucose uptake remained low regardless of PD-1 expression (Figures 2G and 2H). Although the reciprocal relationship between glucose uptake and PD-1 expression was observed in $CD8^+$ T cells from all groups (UI, *Mtb*, BCG), the higher proportion of PD-1^+ cells present during *Mtb* infection suggests that these shifts in metabolism could have greater implications in TB disease. Although we were unable to compare glucose uptake with cytokine production, because of assay incompatibility, we determined that PD-1 expression had little effect on IFN-γ or TNF-α production (Figure S3).

These results confirm findings initially observed in our global gene expression analysis (Figure 1E) and highlight how increased inhibitory receptor expression may affect metabolic reprogramming during *Mtb* infection. The increased proportion of cells expressing PD-1, CTLA-4, and KLRG-1 in total and TB10.4-TET^+ populations in conjunction with decreased glucose uptake suggests that these receptors may participate in metabolic control events that impair the T cell response as chronic *Mtb* infection progresses.

Mitochondrial Respiration Deteriorates in $CD8^+$ T Cells during *Mtb* Infection, Increasing Their Dependency on Glycolysis

To test whether metabolic reprogramming occurs in $CD8^+$ T cells during *Mtb* infection compared with BCG, we used extracellular metabolic flux analysis to measure mitochondrial respiration and extracellular acidification (Figure 3A) following stress induced by mitochondrial inhibition in a cell mito stress test (CMST; see STAR Methods; Figure 3B). On D21, $CD8^+$ T cells from BCG and *Mtb*-infected mice had comparable rates of basal respiration, as indicated by similar oxygen consumption rates (OCRs) and extracellular acidification rates (ECARs) (Figure 3C), CMST profiles (Figure 3D), and maximal respiratory capacity (MRC)/spare respiratory capacity (SRC) values (Figure 3E). Relative to UI mice, infection reduced basal respiration as well as the cells' ability to cope under mitochondrial stress. A bioenergetic phenogram (OCR as a function of ECAR) can be used to illustrate the energy phenotype of a cell. Using this approach, cells can be described as aerobic, energetic, glycolytic, or quiescent. At D21, $CD8^+$ T cells from *Mtb*- and BCG-infected mice displayed an intermediate energy phenotype that was less aerobic and less glycolytic than UI cells (Figure 3F). By D35, basal OCR had decreased in cells from *Mtb*-infected

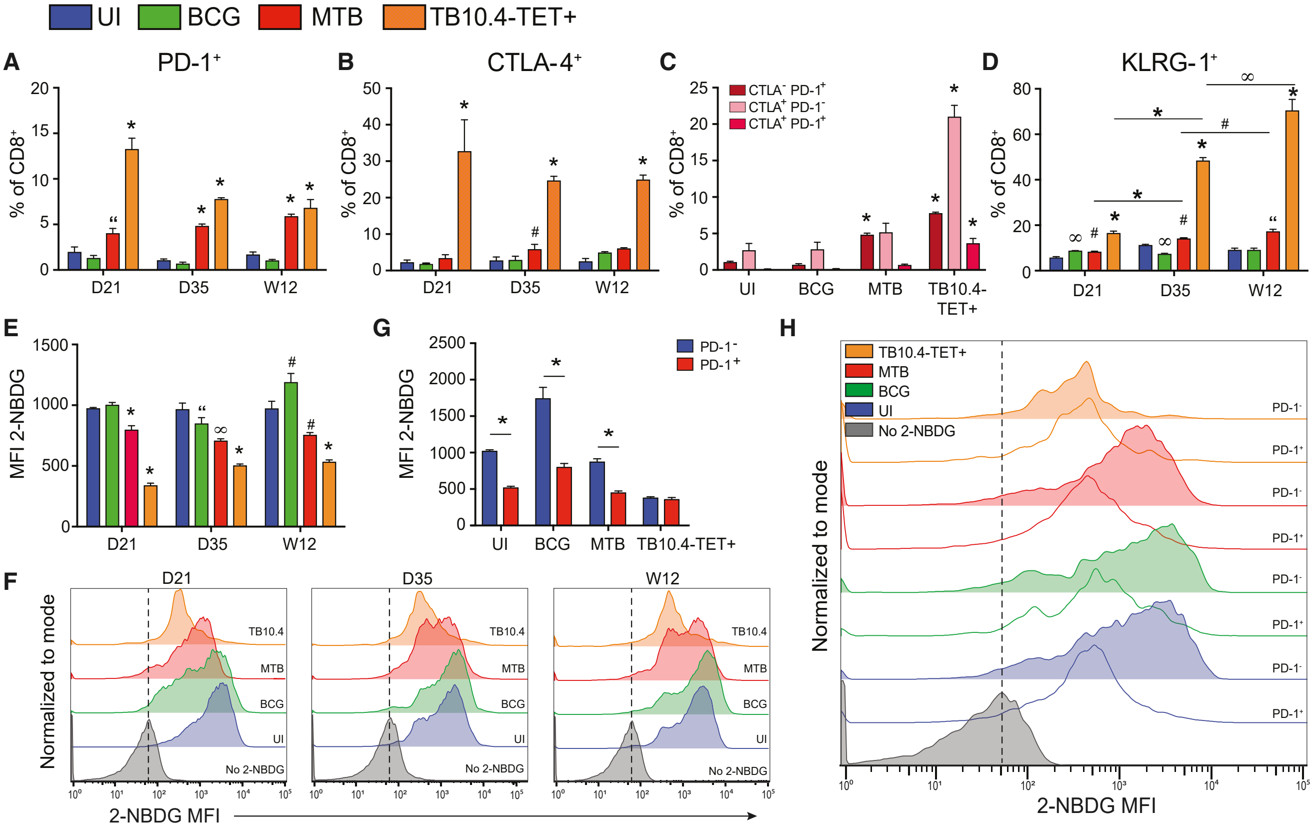

Figure 2. Elevated Inhibitory Receptor Expression Corresponds with Decreased Glucose Uptake in CD8$^+$ T Cells

(A and B) Proportion of total or *Mtb*-specific (TB10.4-TET$^+$) CD8$^+$ T cells expressing (A) PD-1 or (B) CTLA-4 over the course of *Mtb* or BCG infection.

(C) Proportion of cells co-expressing PD-1 and CTLA-4 at 12 weeks post-infection.

(D) Proportion of KLRG-1-expressing cells during infection.

(E and F) Glucose uptake measured by incorporation of the glucose analog 2-NBDG in total or TB10.4-TET$^+$ CD8$^+$ T cells (E) along with representative histograms (F).

(G and H) 2-NBDG uptake in CD8$^+$ T cells expressing (or not expressing) PD-1 (G) with representative histogram (H) at 12 weeks post-infection.

All data represent CD8$^+$ T cells purified from the lungs of uninfected (UI), *Mtb*-infected, or BCG-infected mice; TB10.4-TET$^+$ cells are from *Mtb*-infected mice. 2-NBDG, 2-[*N*-(7-nitrobenz-2-oxa-1,3-diazol-4-yl)amino]-2-deoxyglucose. Statistics, unless otherwise indicated, are relative to UI. Data are representative of three independent experiments (n = 5 mice per group). “$p \leq 0.05$, $^{\#}p \leq 0.01$ $^{\infty}p \leq 0.005$, and $^{*}p \leq 0.001$ by one-way ANOVA or unpaired Student’s t test. Error bars are mean ± SD.

mice, and basal ECAR had increased in both *Mtb* and BCG groups compared with D21 (Figure 3G), although UI cells still maintained higher basal levels of both parameters. The CMST at D35 revealed that T cells from BCG-infected mice performed better under mitochondrial stress than cells from *Mtb*-infected mice (Figure 3H), evidenced by higher MRC/SRC values (Figure 3I); they also displayed a more energetic/glycolytic energy phenotype than cells from *Mtb* infection (Figure 3J). By W12, basal OCR and ECAR were further reduced in cells from *Mtb*-infected mice compared with earlier time points, while basal respiration in the UI and BCG groups stayed consistent with D35 measurements (Figure 3K). By W12, we were able to purify enough TB10.4-TET$^+$ cells to run an additional CMST and compare *Mtb*-specific T cells with total CD8$^+$ cells. Interestingly, TB10.4-TET$^+$ cells at W12 displayed even lower rates of basal respiration (OCR) and ECAR than total CD8$^+$ T cells from *Mtb*-infected mice (Figure 3K). When the CMST profiles (Figure 3L) and all associated parameters (Figure 3M) were compared at W12, distinct differences were observed between *Mtb* and BCG infections. Mitochondrial respiration had deteriorated in CD8$^+$ T cells from mice chronically infected with *Mtb*; these cells had a diminished ability to cope under stress, evidenced by decreased MRC/SRC values (Figure 3M); all other parameters tested were also significantly reduced. Furthermore, all of these effects appeared more pronounced in the *Mtb*-specific TB10.4-TET$^+$ population. By W12, total CD8$^+$ and TB10.4-TET$^+$ T cells from *Mtb*-infected mice displayed “quiescent” energy phenotypes, compared with UI (energetic) and BCG (intermediate) cell populations (Figure 3N). Notably, a similar bioenergetic shift toward this quiescent energy phenotype was observed in *Mtb*-infected macrophages (Cumming et al., 2018). To correlate these bioenergetic shifts with changes in the relative abundance of naive, effector, and memory T cell subsets found in the CD8$^+$ T cell populations from UI,

OPEN ACCESS
CellPress

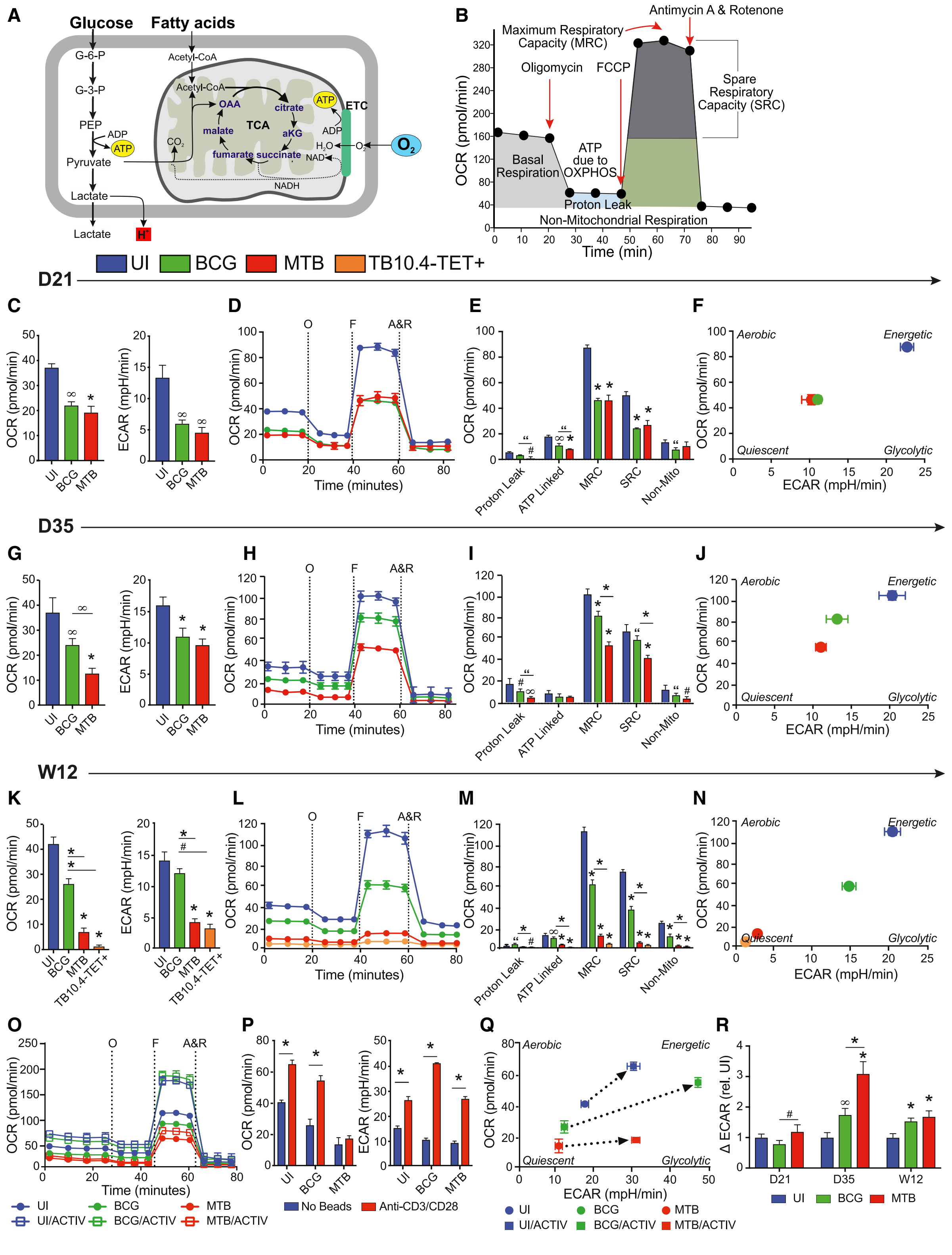

Figure 3. ***Mtb*** **Infection Suppresses Mitochondrial Respiration and Increases Glycolytic Dependency**

(A and B) Aerobic respiration within the mitochondria in the mammalian cell (A) and the cell mito stress test (CMST) profile generated during extracellular flux (XF) analysis (B). XF analysis was performed on purified lung CD8+ T cells during *Mtb* or BCG infection.

(C) Oxygen consumption rate (OCR) and extracellular acidification rate (ECAR) at baseline.

(legend continued on next page)

BCG-infected, and *Mtb*-infected mice, we measured surface expression of CD44 and CD62 and found that the distribution of naive, effector, and memory cells in BCG-infected mice more closely resembled UI mice (Figure S4).

Next we examined the capacity of $CD8^+$ T cells from infected mice to shift their bioenergetic metabolism in response to activation *ex vivo*. CMST profiles generated in the presence or absence of anti-CD3/anti-CD28 activator beads revealed distinct bioenergetic activation capacities among $CD8^+$ T cells from UI, *Mtb*-infected, or BCG-infected mice (Figure 3O). $CD8^+$ T cells from BCG-infected mice were most efficient at ramping up glycolysis (ECAR) and OXPHOS (OCR) upon activation (Figures 3P and 3Q). Although T cells from *Mtb*-infected mice were able to increase ECAR to the same extent as UI cells, their ability to ramp up mitochondrial OXPHOS was impaired (Figures 3P and 3Q).

To investigate whether glycolytic capacity was altered in $CD8^+$-T cells during *Mtb* infection, cells were subjected to a glycolysis stress test (GST). ECAR was measured before and after the addition of glucose (ΔECAR), which indicates how dependent cells are on glucose (and glycolysis) for energy. Under conditions of glucose deprivation, we observed that $CD8^+$ T cells from *Mtb*-infected mice were more dependent on glycolysis than cells from UI or BCG groups at all time points measured, and was highest at D35 (Figure 3R). Intriguingly, these data are distinct from the glucose uptake (2-NBDG) studies (Figures 2E and 2F); these two datasets convey different aspects of glucose uptake and glycolytic dependency. The GST involves a glucose deprivation step, demonstrating the cells' reliance on glucose as a fuel source, while the 2-NBDG study, performed without glucose restriction, provides a better understanding of the cells' ability to take up glucose under basal conditions. Low basal rates of ECAR and OCR (Figures 3C, 3G, and 3K) observed during the CMST corroborate the 2-NBDG studies, suggesting that glucose uptake is low because cell metabolism (OXPHOS and glycolysis) is low. Similar observations were described in T cells from mice chronically infected with LCMV (Bengsch et al., 2016).

T cells can switch their dependency from glucose to fatty acids (FAs) depending on their differentiation state (van der Windt et al., 2013). After mitochondrial uncoupling with carbonyl cyanide-4-[trifluoromethoxy] phenylhydrazone (FCCP), we blocked FA oxidation (FAO) using the Cpt1a inhibitor, etomoxir. We did not observe any differences in FAO dependency in any group (UI, *Mtb*, BCG), indicating that the differences in OCR we observed in earlier experiments were not due to altered FA metabolism (Figure S5).

In sum, these data highlight the presence of distinct bioenergetic signatures in $CD8^+$ T cells from pathogenic and non-pathogenic infections over time. We show that mitochondrial respiration deteriorates in total and *Mtb*-specific $CD8^+$ T cells as *Mtb* infection progresses, promoting a state of metabolic quiescence; this compromised mitochondrial function is also observed upon activation, when cells from *Mtb* infection fail to ramp up OXPHOS to the same extent as cells from UI or BCG groups. To compensate for the decline in OXPHOS, $CD8^+$ T cells from *Mtb*-infected mice shift their dependency toward glycolysis. Unlike *Mtb*, $CD8^+$ T cells from BCG-infected mice regain their respiratory capacity over time. These data provide insights into the possible virulence mechanisms of *Mtb* and demonstrate how metabolic reprogramming could contribute favorably or detrimentally to disease outcome.

Mtb Infection Induces Glycolytic Reprogramming in $CD8^+$ T Cells

To further investigate the increased dependency on glycolysis observed in $CD8^+$ T cells during *Mtb* infection (Figure 3R), we used stable isotope labeling (D-glucose-$^{13}C_6$) to measure the level of ^{13}C incorporation into metabolites involved in glycolysis and the TCA cycle. At both D21 and D35, pyruvate and lactate metabolite pools were significantly more enriched with M+3 isotopologues in T cells from *Mtb*-infected mice compared with UI and BCG metabolite pools (Figure S6A). At W12, only pyruvate was significantly increased in T cells from *Mtb*-infected mice. These results corresponded with elevated ATP and ADP levels at D21 and D35 compared with BCG, although there were fewer differences in the levels of these species compared with UI cells, suggesting that ATP molecules may be generated in similar amounts but through different metabolic pathways in these cells (Figure S6B). Because there was no corresponding increase in OCR linked to ATP production in $CD8^+$ T cells during *Mtb* infection (Figures 3E, 3I, and 3M), these data suggest that the increase in ATP observed is due to residual substrate-level phosphorylation. To determine whether ATP is produced by glycolysis rather than the TCA cycle during *Mtb* infection, we examined the total levels of ^{13}C incorporation in the TCA cycle intermediates citrate and succinate. Total incorporation (%M+) into citrate at D21, D35, and W12 increased in $CD8^+$ T cells during *Mtb* infection relative to cells from UI and BCG-infected mice; however, no differences were observed in succinate (Figure S6C). Increased levels of citrate, but not succinate, suggest that citrate is being shunted back into the cytosol to fuel macromolecular synthesis via acetyl-CoA, a hallmark of T cell activation (Chang et al., 2013). Using this carbon tracing approach, we confirm that *Mtb* infection triggers glycolytic reprogramming in $CD8^+$ T cells, particularly at D35, verifying our bioenergetic analyses (Figure 3R).

Dysfunctional Mitochondria Accumulate in $CD8^+$ T Cells during Chronic *Mtb* Infection

Persistent antigen stimulation during chronic infection perpetuates inflammatory signals that can affect the metabolic health,

(D–N) OCR measured during a CMST (D) with associated bioenergetic parameters (E) and corresponding phenogram after uncoupling with FCCP at D21 post-infection (F), D35 post-infection (G–J), and W12 post-infection (K–N).

(O) CMST profile with/without anti-CD3/anti-CD28 activator beads.

(P and Q) OCR and ECAR at baseline with/without activation (P) and phenogram before/after activation at baseline (Q), performed at D35 post-infection.

(R) Glycolysis, measured by the change in ECAR pre- and post-injection with glucose.

TB10.4-TET^+ cells are from *Mtb*-infected mice. A&R, antimycin A and rotenone; F, carbonyl cyanide-4-[trifluoromethoxy] phenylhydrazone or FCCP; O, oligomycin;. Statistics, unless otherwise indicated, are relative to uninfected (UI). Data are representative of three independent experiments (n = 5 mice per group). “$p \leq 0.05$, $^{\#}p \leq 0.01$, $^{\infty}p \leq 0.005$, and $^{*}p \leq 0.001$ by one-way ANOVA. Error bars are mean ± SD.

function, and lifespan of a cell. An early indicator of T cell exhaustion is the accumulation of dysfunctional mitochondria, characterized by decreased mitochondrial membrane potential ($\Delta\Psi_m$), increased mitochondrial mass (MM), and increased mitochondrial reactive oxygen species (mROS) (Bengsch et al., 2016; Schurich et al., 2016). To investigate whether the decline in OXPHOS observed in CD8$^+$ T cells during *Mtb* infection could be due to an accumulation of dysfunctional mitochondria, we quantified MM and $\Delta\Psi_m$ in CD44$^+$ effector T cells. At D21 post-infection, MM increased in *Mtb*-specific TB10.4-TET$^+$ cells and in total CD44$^+$ CD8$^+$ T cells from BCG-infected mice; this effect was abrogated by D35 in BCG but was sustained in TB10.4-TET$^+$ cells (Figure 4A). MM was significantly higher in total and TB10.4-TET$^+$ cells from *Mtb*-infected mice at W12; this corresponded with a decrease in $\Delta\Psi_m$ that was most pronounced at W12 (Figure 4B). Next, we gated on MTG$^+$MTDR^{low} cells, identifying T cells with depolarized mitochondria (Figure 4C); the proportion of cells with depolarized mitochondria increased over the course of infection in total and TB10.4-TET$^+$ cells from *Mtb*-infected mice as well as in T cells from BCG, although to a lesser extent (Figure 4C). By W12, close to 40% of TB10.4-TET$^+$ cells had depolarized mitochondria.

Mitochondrial ROS has been associated with a loss of $\Delta\Psi_m$ (Willems et al., 2015). *Mtb* infection increased the proportion of MitoSOX$^+$ cells and the signal intensity (mean fluorescence intensity [MFI], corresponding with increased mROS production) over time, particularly in TB10.4-TET$^+$ cells (Figures 4D and 4E). Although it is known that mROS is induced upon T cell activation, high and sustained levels of mROS have been shown to be detrimental to mitochondrial function (Fisicaro et al., 2017).

Next, we investigated whether inhibitory receptor expression corresponded with the accumulation of dysfunctional mitochondria. CD8$^+$ T cells from *Mtb*-infected mice that had the highest MM, lowest $\Delta\Psi_m$ and elevated mROS expressed CTLA-4, alone or in conjunction with PD-1 (Figures 4F–4H). Interestingly, cells expressing PD-1 alone had fewer dysfunctional mitochondria and resembled cells not expressing either inhibitory receptor (CTLA-4$^-$ PD-1$^-$).

Together, these results suggest that chronic *Mtb* infection coincides with an accumulation of effector CD8$^+$ T cells that display increased MM, decreased $\Delta\Psi_m$ and high mROS, all features of dysfunctional mitochondria. This population is particularly enriched in TB10.4-TET$^+$ cells, as well as on cells expressing CTLA-4, suggesting that this receptor may be linked to downstream regulation of mitochondrial metabolism or mitophagy.

Cytokine Production by *Mtb*-Specific CD8$^+$ T Cells Can Be Modulated by Glycolytic Intervention

Metabolic reprogramming is often reflected by changes in downstream effector function (Chang et al., 2013; O'Neill and Hardie, 2013). To identify whether changes in bioenergetics and mitochondrial health reflect downstream effector function, we investigated the impact of *Mtb* infection on cytokine production in CD8$^+$ T cells. IFN-γ production increased in total and TB10.4-TET$^+$ CD44$^+$CD8$^+$ effector T cells from *Mtb*-infected mice compared with CD44$^+$CD8$^+$ T cells from UI and BCG-infected mice at D21 and D35 post-infection (Figure 5A). By W12, the proportion of IFN-γ-producing cells decreased, matching those detected in cells from UI and BCG groups. TNF-α production increased at D35 in total and TB10.4-TET$^+$ T cells from *Mtb*-infected mice and this trend was maintained at W12 (Figure 5B). Interestingly, IL-2 production decreased over time in *Mtb*-infected mice compared with the UI and BCG groups (Figure 5C).

Polyfunctionality is an important characteristic of robust T cell responses that can be lost when effector function declines (Wang et al., 2011). The number of polyfunctional CD44$^+$CD8$^+$ T cells we observed was extremely small in all groups. The percentage of total CD44$^+$CD8$^+$ T cells expressing more than one cytokine was highest in BCG-infected mice at D21 post-infection, while total and TB10.4-TET$^+$ cells from *Mtb*-infected mice demonstrated the highest degree of polyfunctionality at D35 (Figure 5D). Polyfunctionality decreased in all groups at W12; the only notable difference was an increase in the proportion of single cytokine-producing TB10.4-TET$^+$ cells that predominantly produced TNF-α (Figure 5B).

Next, we investigated the metabolic flexibility of CD8$^+$ effector T cells by examining how specific metabolic inhibitors modulated their cytokine production. Inhibiting glycolysis with 2-deoxyglucose (2-DG) revealed that total and TB10.4-TET$^+$ cells from *Mtb*-infected mice were more reliant on glycolysis for IFN-γ production than cells from UI or BCG groups (Figure 5E). Similarly, shifting metabolism from OXPHOS to glycolysis using etomoxir enhanced IFN-γ production in total and TB10.4-TET$^+$ cells from *Mtb*-infected mice to a greater extent than cells from UI or BCG groups (Figure 5F). The lack of cytokine production observed in UI and BCG-infected mice in the presence of etomoxir suggests that these cells may be hardwired to resist glycolytic reprogramming in their current state.

To further investigate the effects of glycolytic reprogramming on polyfunctionality and glucose uptake in TB10.4-TET$^+$ cells, we used metabolic inhibitors in combination with 2-NBDG. Inhibiting glycolysis with 2-DG reduced the frequency of TB10.4-TET$^+$ cells co-expressing IFN-γ and TNF-α, while the same cells treated with etomoxir resulted in an increase in the IFN-γ^+TNF-α^+ subset (Figure 5G). Correspondingly, glucose uptake decreased in TB10.4-TET$^+$ cells exposed to 2-DG, and increased after treatment with etomoxir, except at W12, where 2-NBDG levels appear unaffected by metabolic intervention (Figure 5H).

Here, we identify differences in the timing, quantity, and quality of the cytokine response in CD8$^+$ effector T cells during infection. We show that cytokine production peaks at D35 in TB10.4-TET$^+$ cells, and that this response is dominated by IFN-γ and TNF-α. By W12, IFN-γ production wanes, along with the frequency of polyfunctional cells, and TNF-α producers remain. IL-2 production also decreases over time. Measuring cytokine production in the presence of 2-DG or etomoxir reveals that CD8$^+$ T cells from *Mtb*-infected mice rely more on glycolysis than BCG or UI cells to produce IFN-γ; moreover, cytokine production can be increased or decreased by shifting cell metabolism toward or away from glycolysis. The ability to modulate cytokine responses in this way illustrates how targeted metabolic intervention could be used to beneficially enhance or restrain T cell responses during *Mtb* infection.

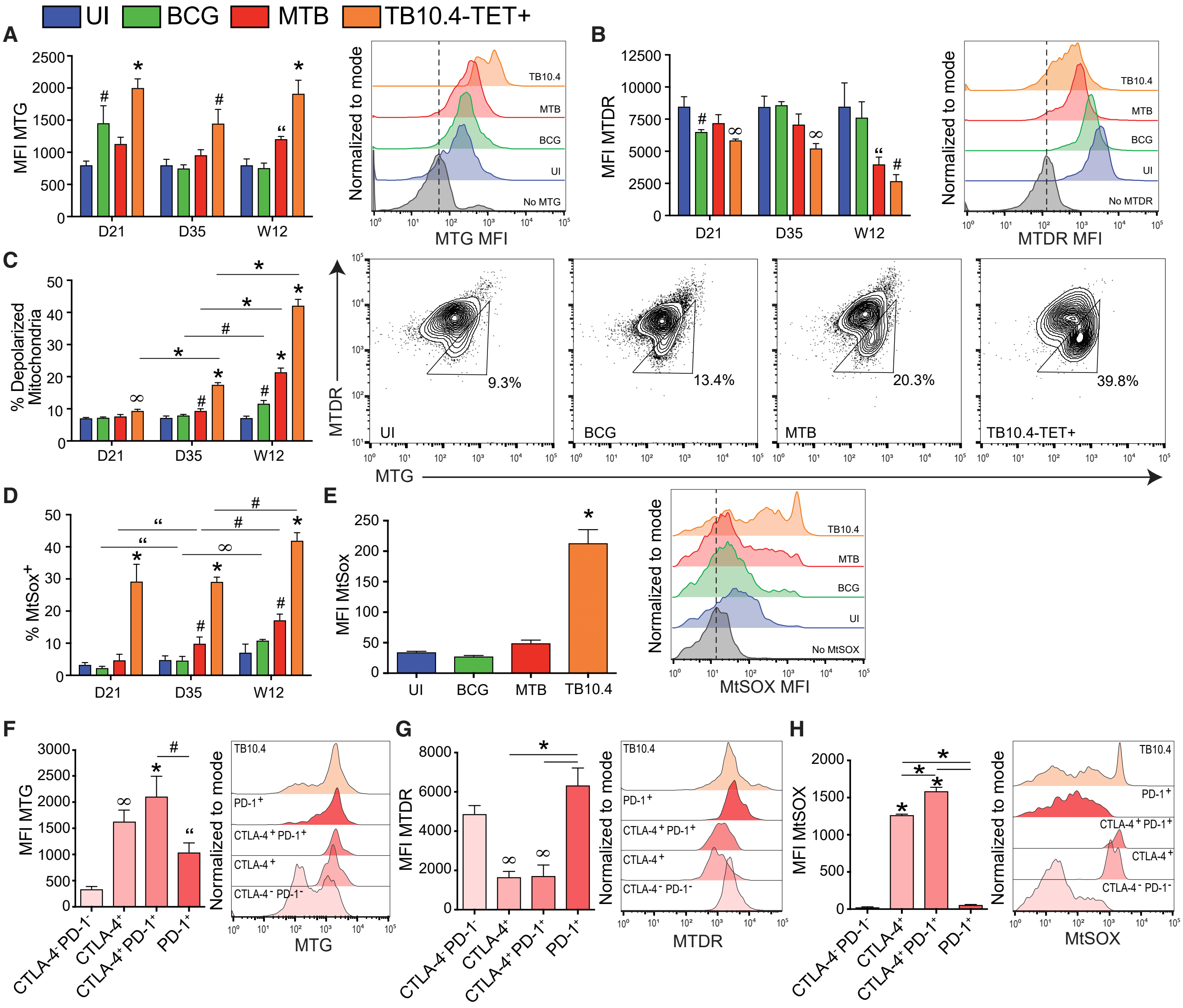

Figure 4. *Mtb* Infection Promotes Accumulation of Dysfunctional Mitochondria and Mitochondrial ROS in TB10.4-TET⁺ Cells

Mitochondrial profiling of total and *Mtb*-specific CD44⁺CD8⁺ T cells in *Mtb*- and BCG-infected mice.

(A and B) Mitochondrial mass, measured by MitoTracker Green (MTG) mean fluorescence intensity (MFI) (A), and mitochondrial membrane potential, measured by MitoTracker Deep Red (MTDR) MFI (B), with representative histograms at W12.

(C) Proportion of $CD44^+CD8^+$ T cells with depolarized mitochondria identified as $MTG^{high}MTDR^{low}$ (triangle gate), with representative plots at W12.

(D) Proportion of $CD44^+CD8^+$ T cells that are $MitoSOX^+$ (MtSox).

(E) MFI of mROS in $CD44^+CD8^+$ T cells and representative histogram at W12.

(F–H) MTG (F), MTDR (G), and mROS (H) in *Mtb*-specific TB10.4-TET^+ cells expressing CTLA-4 and/or PD-1 with representative histograms at D35 post-infection. TB10.4-TET^+ cells are from *Mtb*-infected mice. Statistics, unless otherwise indicated, are relative to uninfected (UI). Data are representative of three independent experiments (N = 5 mice per group). “$p \leq 0.05$, $^{\#}p \leq 0.01$, $^{\infty}p \leq 0.005$, and $^{*}p \leq 0.001$ by one-way ANOVA. Error bars are mean ± SD.

Metabolic Reprogramming by Metformin Alters Effector Function and Rejuvenates Mitochondrial Respiration in $CD8^+$ T Cells during *Mtb* Infection

Metformin (MET) is an existing US Food and Drug Administration (FDA)-approved drug that has been proposed to have therapeutic potential for the effective treatment of TB (Mahon and Hafner, 2015; Singhal et al., 2014; Zumla et al., 2016). When MET is used in combination with anti-*Mtb*-targeting drugs, it can act synergistically to further reduce bacillary burden in mice infected with *Mtb* (Singhal et al., 2014). There are multiple mechanistic targets of MET, including AMPK signaling and GAPDH, but most of these mechanisms point to aspects of mitochondrial biology (Cao et al., 2014; Griss et al., 2015; Meng et al., 2015; Wheaton et al., 2014). We hypothesized that MET could improve the outcome of TB disease by targeting immunometabolic checkpoints required to control infection. To test this hypothesis, we

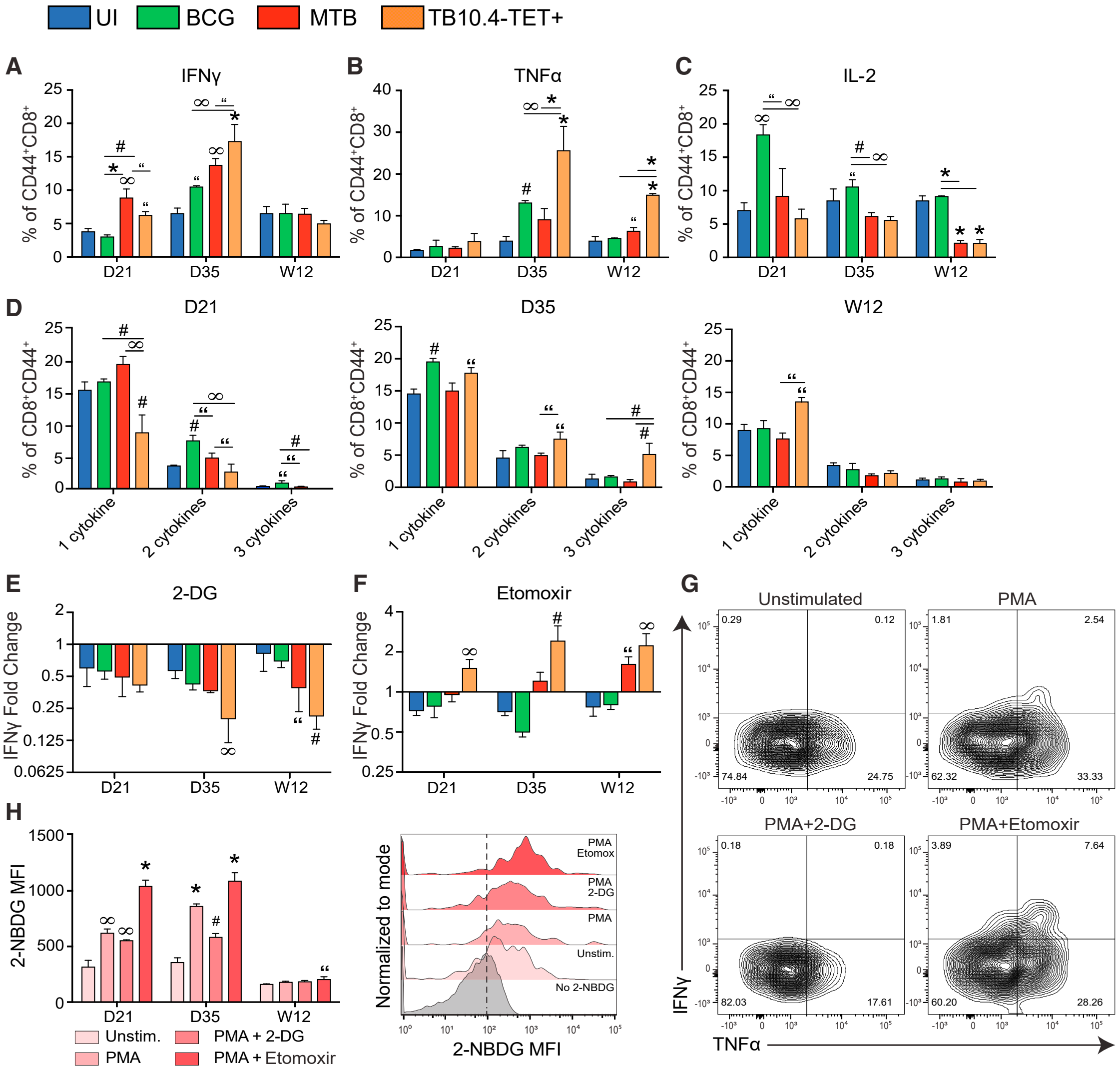

Figure 5. Metabolic Intervention Modulates Cytokine Production in TB10.4-TET+ Cells

(A–C) IFNγ (A), TNFα (B), and IL-2 (C) production in CD44+CD8+ T cells purified from the lungs of uninfected (UI), *Mtb*-infected, or BCG-infected mice after re-stimulation with PMA/ionomycin.

(D) The proportion of polyfunctional T cells within each CD44+CD8+ T cell subset, on the basis of co-expression of IFNγ, TNFα, and/or IL-2 (single, double, or triple combinations).

(E and F) Fold change in IFNγ production following treatment with 2-DG (E) or etomoxir (F) relative to untreated cells re-stimulated with PMA/ionomycin.

(G) Cytokine production by TB10.4-TET+ cells in the presence/absence of 2-DG or etomoxir.

(H) Glucose uptake (2-NBDG MFI) in cells treated with or without PMA/ionomycin with or without 2-DG or etomoxir and representative histogram. Measurements shown in (G) and (H) represent D35 post-infection.

TB10.4-TET+ cells are from *Mtb*-infected mice. 2-DG, 2-deoxyglucose. Statistics, unless otherwise indicated, are relative to UI. Data are representative of three independent experiments (N = 5 mice per group). “$p \leq 0.05$, $^{\#}p \leq 0.01$, $^{\infty}p \leq 0.005$, and $^{*}p \leq 0.001$ by one-way ANOVA. Error bars are mean ± SD.

infected mice with *Mtb*, initiated MET treatment on day 7 post-infection and analyzed the bioenergetic and functional readouts of lung CD8+ T cells at D21 and D35 post-infection (Figure 6A).

Similar to previous studies (Singhal et al., 2014), we did not observe differences in bacillary burden or histopathology at D21 or D35 post-infection with MET treatment alone (Figures 6B and 6C). On D21, CD8+ T cells from MET-treated, *Mtb*-infected mice displayed very few bioenergetic differences relative to their untreated, infected counterparts (Figures 6D–6G), although UI+MET cells did have higher MRC and SRC relative to untreated, UI cells (Figure 6F). However, by D35, relative to untreated, infected mice, CD8+ T cells from MET-treated, *Mtb*-infected mice displayed higher basal OCR (Figure 6H) and an increased capacity to withstand mitochondrial stress (Figure 6I), evidenced by enhanced MRC and SRC (Figure 6J). The improved bioenergetics were best illustrated in the energy

phenogram, whereby cells from *Mtb*-infected mice clearly shift from quiescent to energetic energy phenotypes after MET treatment (Figure 6K). Next, we identified whether MET influenced the cells' ability to use glucose as fuel. Glycolysis was reduced by MET treatment (ΔECAR) in *Mtb*-infected mice at D21 and D35 post-infection (Figure 6L), suggesting that T cells from MET-treated mice are less dependent on glycolysis for energy production, likely because of the restoration of OXPHOS.

Flow cytometry on $CD8^+$ T cells from the lungs and spleen revealed a 2-fold increase in the *Mtb*-specific TB10.4-TET^+ population following MET treatment (Figure 6M). Interestingly, these *Mtb*-specific cells produced fewer cytokines than their untreated counterparts, evidenced by decreased IL-2 at D21, IFN-γ at D35, and TNF-α at both time points (Figure 6N). MET treatment also decreased the frequency of polyfunctional T cells at D35 (Figure 6O).

In sum, these data highlight MET's ability to alter *Mtb*-specific $CD8^+$ T cell responses *in vivo*. MET enhanced the number of TB10.4-TET^+ cells at local and systemic sites during infection, decreased inflammatory cytokine production, most notably TNF-α, and improved mitochondrial bioenergetics in these cells at D35 post-infection. As anticipated, these MET-mediated effects were not unique to infection, as shifts were also observed in UI mice treated with MET, although the effects were smaller in magnitude. These data suggest that MET can reverse the quiescent metabolic state observed in $CD8^+$ T cells during chronic *Mtb* infection, rejuvenate OXPHOS, and ultimately enhance the effectiveness of *Mtb*-specific $CD8^+$ T cell responses.

DISCUSSION

Metabolism has been largely overlooked as a key driver of the suboptimal immune responses generated during chronic *Mtb* infection. In the present study, we examined the immunometabolic signatures of $CD8^+$ T cells from the lungs of mice infected with *Mtb* or the non-pathogenic vaccine strain, BCG, and provide insight into how metabolism may play an important role in the development of protective versus pathogenic immunity. We show that *Mtb* infection corresponds with the metabolic and functional deterioration of $CD8^+$ T cells over time, evidenced by the presence of an *Mtb*-specific T cell population displaying significant bioenergetic insufficiencies, declining mitochondrial health, and limited cytokine production, all early indicators of T cell exhaustion. Bioenergetic analyses revealed a progressive decline in mitochondrial respiration as *Mtb* infection progressed that decreased the cells' ability to cope under stress, eventually forcing the cell into a state of metabolic quiescence. This resulted in an increased dependency on residual glycolysis that could be confirmed by carbon tracer studies. Impairment of OXPHOS was also observed in $CD8^+$ T cells from *Mtb*-infected mice in response to external activation signals. Furthermore, this reduction in OXPHOS correlated with an increase in markers associated with dysfunctional mitochondria, particularly in TB10.4-TET^+ cells. Cells that had the highest proportion of dysfunctional mitochondria expressed CTLA-4 alone or in conjunction with PD-1, suggesting that specific immune checkpoint regulators may play important roles in driving this phenotype. Intriguingly, the deterioration of mitochondrial metabolism and elevated markers of mitochondrial dysfunction were not observed in BCG infection. Cytokine production also declined over time in TB10.4-TET^+ cells, evidenced by a decrease in the quantity and quality of cytokine-producing cells detected at W12. Notably, metabolic intervention was able to modulate the metabolic and functional capacities of $CD8^+$ T cells from *Mtb*-infected mice. In summary, we have identified an immunometabolic signature unique to *Mtb* infection that could be exploited by pharmacologic agents that target key energy pathways to improve immunological control of *Mtb*.

Incorrect metabolic remodeling contributes to the breakdown of protective immunity in numerous disease states, including chronic viral infections and cancer (Bengsch et al., 2016; Ho et al., 2015; Schurich et al., 2016; Siska et al., 2017). Many similarities exist between what we have observed during chronic *Mtb* infection and what has been described in early exhausted T cells isolated from mice during chronic LCMV infection (Bengsch et al., 2016). However, we did not detect increased FAO in the $CD8^+$ T cell population during chronic *Mtb* infection; instead, these cells had an increased dependency on glycolysis that was observed only upon glucose deprivation and not under basal conditions. Exhausted HBV-specific $CD8^+$ T cells from virus-infected individuals display similar increases in glycolysis, which is thought to compensate for the decrease in mitochondrial function observed (Schurich et al., 2016). During *Mtb* infection, we observed the largest shift in glycolysis at D35 post-infection, which also corresponded with the highest levels of IFN-γ and TNF-α production. When metabolic inhibitors were used to alter glycolytic reprogramming (2-DG, etomoxir), we observed greater shifts in cytokine production in $CD8^+$ T cells from *Mtb*-infected mice compared with UI and BCG groups. These data suggest that *Mtb* infection conditions T cells to rely more on glycolysis for energy production than cells from UI or BCG-infected mice. Glycolytic reprogramming is part of normal T cell activation, but we would expect these cells to have elevated basal ECAR and display a glycolytic energy phenotype compared with UI cells. Instead, it appears that these cells are relying on glycolysis to compensate for increasing mitochondrial dysfunction, particularly at later stages of infection. The T cell activation assay supported the latter conclusion, evidenced by the fact that T cells from *Mtb*-infected mice were unable to ramp up OXPHOS in response to activation signals. Together, these data shed light on the bioenergetic impairments acquired by $CD8^+$ T cells during chronic *Mtb* infection. It will be important to investigate whether similar bioenergetic impairments exist in $CD4^+$ T cells, especially because there are important interactions between these two T cell subsets in TB lesions, however, this will be the focus of a future study. With the knowledge that metabolic dysfunction can affect the differentiation, function, and fitness of a cell long term, selectively targeting OXPHOS or glycolysis in these cells could represent an opportunity to improve control of *Mtb* by boosting *Mtb*-specific T cell responses, an approach that is already being explored in cancer (Sukumar et al., 2013).

Although glycolysis is necessary for T cell activation and the generation of effector proteins (MacIver et al., 2013), it is not always beneficial to the cell. Persistent antigen stimulation during

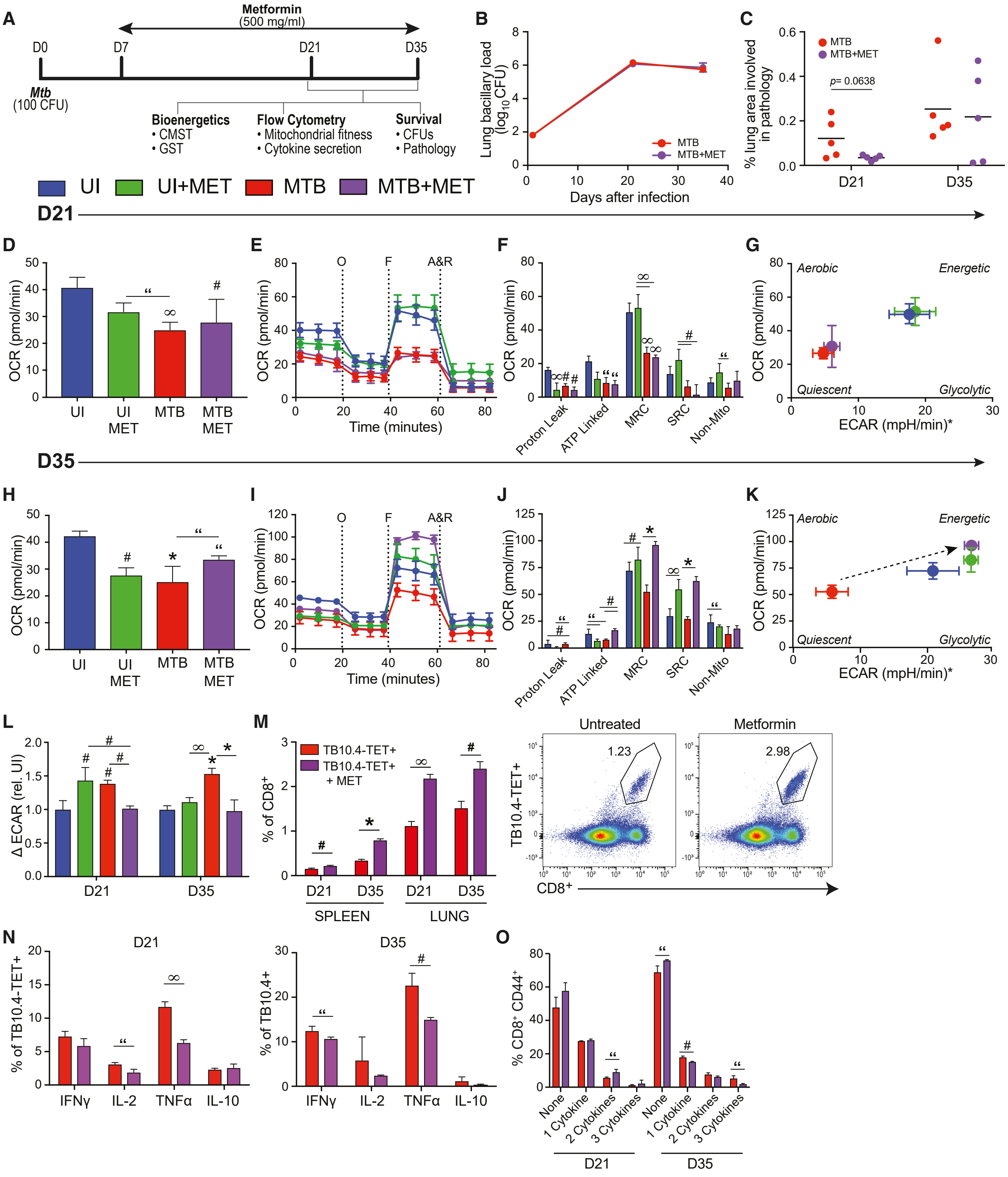

Figure 6. Metformin Rejuvenates OXPHOS and Reduces Inflammatory Cytokine Production in TB10.4-TET^{+} Cells

(A) Experimental timeline.

(B and C) CFUs in the lungs of mice treated with or without metformin (MET) over the course of infection (B) and corresponding pathology scores performed by morphometric analysis (Visiopharm) on lung tissue sections stained with H&E (C). Total CD8^{+} T cells purified from the lungs of uninfected (UI) or *Mtb*-infected mice treated with or without MET were analyzed using XF or flow cytometry.

(legend continued on next page)

chronic infection perpetuates inflammatory signals and increases the energy demands on a cell, promoting dysfunctional states like exhaustion. T cell activation and effector-related signaling pathways were significantly upregulated in $CD8^+$ T cells during *Mtb* infection compared with BCG at D21, accompanied by genes associated with apoptosis and exhaustion at W12, suggesting that *Mtb* infection supports an inflammatory state that cannot be sustained long term. Consistent with these findings, we observed an increased proportion of $CD8^+$ effector T cells expressing PD-1 and CTLA-4, and the terminal differentiation marker KLRG-1, during *Mtb* infection. KLRG-1 was expressed on ~80% of TB10.4-TET^+ cells by W12. Elevated levels of KLRG-1 may be responsible for the lack of $CD8^+$ memory precursors generated during *Mtb* infection. Memory T cells rely heavily on mitochondria-derived ATP (van der Windt et al., 2012, 2013); the reverse was observed in $CD8^+$ T cells during *Mtb* infection. This suggests that there is insufficient bioenergetic capacity to support memory development, although we did not look specifically at memory markers in this study. Interestingly, AMPK, an important upstream signaling molecule of mTOR, is a metabolic switch that plays a key role in $CD8^+$ T cell memory development during infection (Rolf et al., 2013) and cancer (Eikawa et al., 2015). AMPK is one of the mechanistic targets of MET, which has been successful at reversing the phenotype of exhausted TILs in cancer immunotherapy (Eikawa et al., 2015) and could have similar therapeutic implications in TB.

Targeting metabolic pathways that regulate effector T cell responses can reinvigorate immunity. The anti-diabetic drug MET has been identified as a potential anti-cancer agent and, most recently, a candidate host-directed therapy for TB (Mahon and Hafner, 2015; Singhal et al., 2014; Zumla et al., 2016). Although we did not observe a reduction in *Mtb* CFUs or pathology with MET treatment, we saw remarkable improvements in $CD8^+$ T cell bioenergetics at D35 post-infection that corresponded with a decreased inflammatory profile. Reduced TNF-α production was particularly relevant because TNF-α has been strongly linked to granuloma formation (Lukacs et al., 1994); similarly, monofunctional, TNF-α-secreting T cells were the strongest predictor of active TB disease in human patients (Harari et al., 2011). MET has previously been shown to decrease inflammatory gene expression in the lungs of *Mtb*-infected mice (Singhal et al., 2014), further validating our findings. We postulate that improved $CD8^+$ T cell immunometabolism may not have coincided with an immediate effect on bacterial growth because *Mtb* is a slow-growing pathogen (24 h doubling time), and pathology takes time to develop (and recede) in this model; therefore, MET may have a more measurable impact long term. Because the effects of chronic inflammation and metabolic remodeling were most pronounced at W12 in our initial studies, MET could reduce immunopathology and CFUs in the lung at later time points (e.g., W12 post-infection). Investigating the long-term effects of MET on *Mtb* infection remains the focus of a separate study. It is also possible that MET enhanced innate immune control of *Mtb* (e.g., phagocytosis, autophagy, phagolysosome function, antigen presentation), thus indirectly altering $CD8^+$ T cell immunometabolism. It will be important to investigate the timing of MET administration, as well as the interactions between immune cell populations over the course of treatment to gain a more in-depth understanding of the mechanisms involved and the clinical implications.

The data presented here represent a significant advancement in the TB field because the results highlight important associations between metabolism and the breakdown of *Mtb* immunity. However, we recognize that further studies are required to delineate whether these observations are the cause or consequence of *Mtb* persistence. Similar to other studies in this interdisciplinary field (Bengsch et al., 2016; Schurich et al., 2016), we analyzed the immunometabolic effects of two clinically distinct infections (*Mtb* versus BCG). Given the study design, it is difficult to pinpoint whether the observed differences are due to different infection kinetics (i.e., pathogen burden, immunopathology) or other virulence mechanisms specific to the pathogen; it is likely a combination thereof. Titrating the infectious dose of BCG so that equivalent numbers of *Mtb* and BCG can be compared at each experimental time point, as demonstrated by Grace and Ernst (2016), may control for some of these variables and should be the focus of a future study. Furthermore, without investigating the interaction between innate immune cells and T cells, it is difficult to establish whether dysregulated T cell immunometabolism is a direct or indirect effect of *Mtb* pathogenesis (i.e., mechanisms aimed at subverting innate immunity could have downstream effects on T cell immunity). Previously, we have shown that *Mtb* decelerates macrophage energy metabolism to support its survival *in vitro* (Cumming et al., 2018), suggesting that innate and adaptive immunometabolism in TB are linked. Although more mechanistic studies are required to address these questions, these data introduce methodologies and workflows that could be used to improve TB drug discovery and evaluate vaccine efficacy in experimental animal models.

Metabolism represents a key node of regulation in T cell function that can be manipulated by microbial pathogens to enhance or temper immunity. Our findings suggest that the metabolic deceleration of host $CD8^+$ T cells may be an underlying mechanism of *Mtb* pathogenesis that leads to the loss of control and eventual breakdown of protective immunity observed in chronic

(D) OCR at baseline.
(E–K) OCR measured during a cell mito stress test (CMST) (E) with associated bioenergetic parameters (F) and corresponding phenogram (G) after uncoupling with FCCP at D21 post-infection and (H–K) D35 post-infection.
(L) Glycolysis, measured by the change in ECAR pre- and post-injection with glucose.
(M) Frequency of *Mtb*-specific, TB10.4-TET^+ cells in lungs and spleen after MET.
(N) Cytokine production in TB10.4-TET^+ cells after re-stimulation with PMA/ionomycin.
(O) Polyfunctionality on the basis of co-expression of IFNγ, TNF-α, and IL-2 (single, double, or triple combinations) of TB10.4-TET^+ cells in (N).
A&R, antimycin A and rotenone; F, carbonyl cyanide-4-[trifluoromethoxy] phenylhydrazone or FCCP; O, oligomycin. *ECAR values were normalized to a zero baseline. Statistics, unless otherwise indicated, are relative to UI. Data are representative of two independent experiments (N = 5 mice per group). “$p \leq 0.05$, $^{\#}p \leq 0.01$, $^{\infty}p \leq 0.005$, and $^{*}p \leq 0.001$ by one-way ANOVA or unpaired Student’s t test. Error bars are mean ± SD.

Mtb infection. Furthermore, we demonstrate how metabolic intervention can improve the metabolic flexibility of these cells and fine-tune effector responses, although these effects require more in-depth study to determine whether they improve *Mtb* control long term. Developing metabolically targeted immunotherapies for TB could boost productive effector T cell responses at the site of infection and improve the fitness of circulating memory T cell populations; they could be used in combination with existing anti-TB treatment or developed to vastly improve the outcome of multidrug-resistant TB. Ultimately, by understanding how metabolism and immunity are linked in TB disease, it may be possible to identify, target, and reverse key immunometabolic checkpoints in T cell-mediated immunity to promote *Mtb* clearance and bolster resistance to reinfection.

STAR★METHODS

Detailed methods are provided in the online version of this paper and include the following:

- KEY RESOURCES TABLE
- LEAD CONTACT AND MATERIALS AVAILABILITY
- EXPERIMENTAL MODEL AND SUBJECT DETAILS
  - Mice
  - Bacteria
- METHOD DETAILS
  - Study design
  - Bacterial infections and drug treatment
  - Bacterial enumeration and T cell isolation
  - RNA preparation, sequencing, and analysis
  - Gene set enrichment analysis
  - Metabolic extracellular flux analysis
  - MHC tetramers, antibodies, and flow cytometry
  - Carbon Tracing, Metabolite Extraction and Mass Spectrometry Analysis
- QUANTIFICATION AND STATISTICAL ANALYSIS
- DATA AND CODE AVAILABILITY

SUPPLEMENTAL INFORMATION

Supplemental Information can be found online at https://doi.org/10.1016/j.celrep.2019.11.034.

ACKNOWLEDGMENTS

We thank Dr. Bridgette Cumming for her XF expertise, Dr. John Adamson and Katya Govender for their LC-MS expertise, and the Africa Health Research Institute (AHRI) Animal Core Facility for ensuring expert animal care. This work was supported by National Institutes of Health (NIH) grants R01AI111940, R61AI138280, and R21A127182 and Bill and Melinda Gates Foundation award OPP1130017 (A.J.C.S.), and was co-funded by CRDF Global and the South African Medical Research Council (A.J.C.S.).

AUTHOR CONTRIBUTIONS

Study Concept and Design, S.L.R. and A.J.C.S.; Data Acquisition, S.L.R., T.M., T.T.J., V.N., and K.W.A.; Data Analysis, S.L.R., D.A.L., D.K.C., and G.W.; Animal Care and Processing, C.M. and B.N.; Manuscript Preparation, S.L.R. and A.J.C.S.

DECLARATION OF INTERESTS

The authors declare no conflicts of interest.

Received: May 28, 2019
Revised: August 19, 2019
Accepted: November 7, 2019
Published: December 10, 2019

REFERENCES

Anders, S., Pyl, P.T., and Huber, W. (2015). HTSeq—a Python framework to work with high-throughput sequencing data. Bioinformatics *31*, 166–169.

Bengsch, B., Johnson, A.L., Kurachi, M., Odorizzi, P.M., Pauken, K.E., Attanasio, J., Stelekati, E., McLane, L.M., Paley, M.A., Delgoffe, G.M., and Wherry, E.J. (2016). Bioenergetic insufficiencies due to metabolic alterations regulated by the inhibitory receptor PD-1 are an early driver of CD8(+) T cell exhaustion. Immunity *45*, 358–373.

Blackburn, S.D., Shin, H., Haining, W.N., Zou, T., Workman, C.J., Polley, A., Betts, M.R., Freeman, G.J., Vignali, D.A., and Wherry, E.J. (2009). Coregulation of CD8+ T cell exhaustion by multiple inhibitory receptors during chronic viral infection. Nat. Immunol. *10*, 29–37.

Buck, M.D., O'Sullivan, D., and Pearce, E.L. (2015). T cell metabolism drives immunity. J. Exp. Med. *212*, 1345–1360.

Cao, J., Meng, S., Chang, E., Beckwith-Fickas, K., Xiong, L., Cole, R.N., Radovick, S., Wondisford, F.E., and He, L. (2014). Low concentrations of metformin suppress glucose production in hepatocytes through AMP-activated protein kinase (AMPK). J. Biol. Chem. *289*, 20435–20446.

Carpenter, S.M., Nunes-Alves, C., Booty, M.G., Way, S.S., and Behar, S.M. (2016). A higher activation threshold of memory CD8+ T cells has a fitness cost that is modified by TCR affinity during tuberculosis. PLoS Pathog. *12*, e1005380.

Chang, C.H., Curtis, J.D., Maggi, L.B., Jr., Faubert, B., Villarino, A.V., O'Sullivan, D., Huang, S.C., van der Windt, G.J., Blagih, J., Qiu, J., et al. (2013). Posttranscriptional control of T cell effector function by aerobic glycolysis. Cell *153*, 1239–1251.

Chen, C.Y., Huang, D., Wang, R.C., Shen, L., Zeng, G., Yao, S., Shen, Y., Halliday, L., Fortman, J., McAllister, M., et al. (2009). A critical role for CD8 T cells in a nonhuman primate model of tuberculosis. PLoS Pathog. *5*, e1000392.

Crespo, J., Sun, H., Welling, T.H., Tian, Z., and Zou, W. (2013). T cell anergy, exhaustion, senescence, and stemness in the tumor microenvironment. Curr. Opin. Immunol. *25*, 214–221.

Cumming, B.M., Addicott, K.W., Adamson, J.H., and Steyn, A.J. (2018). *Mycobacterium tuberculosis* induces decelerated bioenergetic metabolism in human macrophages. eLife *7*, e39169.

Dobin, A., Davis, C.A., Schlesinger, F., Drenkow, J., Zaleski, C., Jha, S., Batut, P., Chaisson, M., and Gingeras, T.R. (2013). STAR: ultrafast universal RNA-seq aligner. Bioinformatics *29*, 15–21.

Dooley, K.E., and Chaisson, R.E. (2009). Tuberculosis and diabetes mellitus: convergence of two epidemics. Lancet Infect. Dis. *9*, 737–746.

Eikawa, S., Nishida, M., Mizukami, S., Yamazaki, C., Nakayama, E., and Udono, H. (2015). Immune-mediated antitumor effect by type 2 diabetes drug, metformin. Proc. Natl. Acad. Sci. U S A *112*, 1809–1814.

Einarsdottir, T., Lockhart, E., and Flynn, J.L. (2009). Cytotoxicity and secretion of gamma interferon are carried out by distinct CD8 T cells during Mycobacterium tuberculosis infection. Infect. Immun. *77*, 4621–4630.

Fine, P.E. (1995). Bacille Calmette-Guérin vaccines: a rough guide. Clin. Infect. Dis. *20*, 11–14.

Fisicaro, P., Barili, V., Montanini, B., Acerbi, G., Ferracin, M., Guerrieri, F., Salerno, D., Boni, C., Massari, M., Cavallo, M.C., et al. (2017). Targeting mitochondrial dysfunction can restore antiviral activity of exhausted HBV-specific CD8 T cells in chronic hepatitis B. Nat. Med. *23*, 327–336.

Flynn, J.L., Goldstein, M.M., Triebold, K.J., Koller, B., and Bloom, B.R. (1992). Major histocompatibility complex class I-restricted T cells are required for resistance to Mycobacterium tuberculosis infection. Proc. Natl. Acad. Sci. U S A *89*, 12013–12017.

Gideon, H.P., Phuah, J., Myers, A.J., Bryson, B.D., Rodgers, M.A., Coleman, M.T., Maiello, P., Rutledge, T., Marino, S., Fortune, S.M., et al. (2015). Variability in tuberculosis granuloma T cell responses exists, but a balance of pro- and anti-inflammatory cytokines is associated with sterilization. PLoS Pathog. *11*, e1004603.

Grace, P.S., and Ernst, J.D. (2016). Suboptimal antigen presentation contributes to virulence of Mycobacterium tuberculosis in vivo. J. Immunol. *196*, 357–364.

Griss, T., Vincent, E.E., Egnatchik, R., Chen, J., Ma, E.H., Faubert, B., Viollet, B., DeBerardinis, R.J., and Jones, R.G. (2015). Metformin antagonizes cancer cell proliferation by suppressing mitochondrial-dependent biosynthesis. PLoS Biol. *13*, e1002309.

Harari, A., Rozot, V., Bellutti Enders, F., Perreau, M., Stalder, J.M., Nicod, L.P., Cavassini, M., Calandra, T., Blanchet, C.L., Jaton, K., et al. (2011). Dominant TNF-α+ Mycobacterium tuberculosis-specific CD4+ T cell responses discriminate between latent infection and active disease. Nat. Med. *17*, 372–376.

Ho, P.C., Bihuniak, J.D., Macintyre, A.N., Staron, M., Liu, X., Amezquita, R., Tsui, Y.C., Cui, G., Micevic, G., Perales, J.C., et al. (2015). Phosphoenolpyruvate is a metabolic checkpoint of anti-tumor T cell responses. Cell *162*, 1217–1228.

Jayaraman, P., Jacques, M.K., Zhu, C., Steblenko, K.M., Stowell, B.L., Madi, A., Anderson, A.C., Kuchroo, V.K., and Behar, S.M. (2016). TIM3 mediates T cell exhaustion during Mycobacterium tuberculosis infection. PLoS Pathog. *12*, e1005490.

Jeon, C.Y., and Murray, M.B. (2008). Diabetes mellitus increases the risk of active tuberculosis: a systematic review of 13 observational studies. PLoS Med. *5*, e152.

Kramer, P.A., Chacko, B.K., Ravi, S., Johnson, M.S., Mitchell, T., and Darley-Usmar, V.M. (2014). Bioenergetics and the oxidative burst: protocols for the isolation and evaluation of human leukocytes and platelets. J. Vis. Exp. (*85*)

Lamprecht, D.A., Finin, P.M., Rahman, M.A., Cumming, B.M., Russell, S.L., Jonnala, S.R., Adamson, J.H., and Steyn, A.J. (2016). Turning the respiratory flexibility of Mycobacterium tuberculosis against itself. Nat. Commun. *7*, 12393.

Lee, J.Y., Jeong, I., Joh, J.S., Jung, Y.W., Sim, S.Y., Choi, B., Jee, H.G., and Lim, D.G. (2015). Differential expression of CD57 in antigen-reactive CD4+ T cells between active and latent tuberculosis infection. Clin. Immunol. *159*, 37–46.

Lönnroth, K., Castro, K.G., Chakaya, J.M., Chauhan, L.S., Floyd, K., Glaziou, P., and Raviglione, M.C. (2010). Tuberculosis control and elimination 2010-50: cure, care, and social development. Lancet *375*, 1814–1829.

Love, M.I., Huber, W., and Anders, S. (2014). Moderated estimation of fold change and dispersion for RNA-seq data with DESeq2. Genome Biol. *15*, 550.

Lukacs, N.W., Chensue, S.W., Strieter, R.M., Warmington, K., and Kunkel, S.L. (1994). Inflammatory granuloma formation is mediated by TNF-alpha-inducible intercellular adhesion molecule-1. J. Immunol. *152*, 5883–5889.

MacIver, N.J., Michalek, R.D., and Rathmell, J.C. (2013). Metabolic regulation of T lymphocytes. Annu. Rev. Immunol. *31*, 259–283.

MacLean, B., Tomazela, D.M., Shulman, N., Chambers, M., Finney, G.L., Frewen, B., Kern, R., Tabb, D.L., Liebler, D.C., and MacCoss, M.J. (2010). Skyline: an open source document editor for creating and analyzing targeted proteomics experiments. Bioinformatics *26*, 966–968.

Mahon, R.N., and Hafner, R. (2015). Immune cell regulatory pathways unexplored as host-directed therapeutic targets for Mycobacterium tuberculosis: an opportunity to apply precision medicine innovations to infectious diseases. Clin. Infect. Dis. *61* (*Suppl 3*), S200–S216.

Meng, S., Cao, J., He, Q., Xiong, L., Chang, E., Radovick, S., Wondisford, F.E., and He, L. (2015). Metformin activates AMP-activated protein kinase by promoting formation of the $\alpha\beta\gamma$ heterotrimeric complex. J. Biol. Chem. *290*, 3793–3802.

Nunes-Alves, C., Booty, M.G., Carpenter, S.M., Jayaraman, P., Rothchild, A.C., and Behar, S.M. (2014). In search of a new paradigm for protective immunity to TB. Nat. Rev. Microbiol. *12*, 289–299.

O'Neill, L.A., and Hardie, D.G. (2013). Metabolism of inflammation limited by AMPK and pseudo-starvation. Nature *493*, 346–355.

Orme, I.M. (1999). Beyond BCG: the potential for a more effective TB vaccine. Mol. Med. Today *5*, 487–492.

Patsoukis, N., Bardhan, K., Chatterjee, P., Sari, D., Liu, B., Bell, L.N., Karoly, E.D., Freeman, G.J., Petkova, V., Seth, P., et al. (2015). PD-1 alters T-cell metabolic reprogramming by inhibiting glycolysis and promoting lipolysis and fatty acid oxidation. Nat. Commun. *6*, 6692.

Rolf, J., Zarrouk, M., Finlay, D.K., Foretz, M., Viollet, B., and Cantrell, D.A. (2013). AMPKα1: a glucose sensor that controls CD8 T-cell memory. Eur. J. Immunol. *43*, 889–896.

Sakai, S., Kauffman, K.D., Sallin, M.A., Sharpe, A.H., Young, H.A., Ganusov, V.V., and Barber, D.L. (2016). CD4 T cell-derived IFN-γ plays a minimal role in control of pulmonary Mycobacterium tuberculosis infection and must be actively repressed by PD-1 to prevent lethal disease. PLoS Pathog. *12*, e1005667.

Schurich, A., Pallett, L.J., Jajbhay, D., Wijngaarden, J., Otano, I., Gill, U.S., Hansi, N., Kennedy, P.T., Nastouli, E., Gilson, R., et al. (2016). Distinct metabolic requirements of exhausted and functional virus-specific CD8 T cells in the same host. Cell Rep. *16*, 1243–1252.

Singh, A., Mohan, A., Dey, A.B., and Mitra, D.K. (2017). Programmed death-1$^+$ T cells inhibit effector T cells at the pathological site of miliary tuberculosis. Clin. Exp. Immunol. *187*, 269–283.

Singhal, A., Jie, L., Kumar, P., Hong, G.S., Leow, M.K., Paleja, B., Tsenova, L., Kurepina, N., Chen, J., Zolezzi, F., et al. (2014). Metformin as adjunct antituberculosis therapy. Sci. Transl. Med. *6*, 263ra159.

Siska, P.J., Beckermann, K.E., Mason, F.M., Andrejeva, G., Greenplate, A.R., Sendor, A.B., Chiang, Y.J., Corona, A.L., Gemta, L.F., Vincent, B.G., et al. (2017). Mitochondrial dysregulation and glycolytic insufficiency functionally impair CD8 T cells infiltrating human renal cell carcinoma. JCI Insight *2*, 93411.

Stenger, S., Hanson, D.A., Teitelbaum, R., Dewan, P., Niazi, K.R., Froelich, C.J., Ganz, T., Thoma-Uszynski, S., Melián, A., Bogdan, C., et al. (1998). An antimicrobial activity of cytolytic T cells mediated by granulysin. Science *282*, 121–125.

Sukumar, M., Liu, J., Ji, Y., Subramanian, M., Crompton, J.G., Yu, Z., Roychoudhuri, R., Palmer, D.C., Muranski, P., Karoly, E.D., et al. (2013). Inhibiting glycolytic metabolism enhances CD8+ T cell memory and antitumor function. J. Clin. Invest. *123*, 4479–4488.

Tameris, M.D., Hatherill, M., Landry, B.S., Scriba, T.J., Snowden, M.A., Lockhart, S., Shea, J.E., McClain, J.B., Hussey, G.D., Hanekom, W.A., et al.; MVA85A 020 Trial Study Team (2013). Safety and efficacy of MVA85A, a new tuberculosis vaccine, in infants previously vaccinated with BCG: a randomised, placebo-controlled phase 2b trial. Lancet *381*, 1021–1028.

van der Windt, G.J., Everts, B., Chang, C.H., Curtis, J.D., Freitas, T.C., Amiel, E., Pearce, E.J., and Pearce, E.L. (2012). Mitochondrial respiratory capacity is a critical regulator of CD8+ T cell memory development. Immunity *36*, 68–78.

van der Windt, G.J., O'Sullivan, D., Everts, B., Huang, S.C., Buck, M.D., Curtis, J.D., Chang, C.H., Smith, A.M., Ai, T., Faubert, B., et al. (2013). CD8 memory T cells have a bioenergetic advantage that underlies their rapid recall ability. Proc. Natl. Acad. Sci. U S A *110*, 14336–14341.

van Pinxteren, L.A., Cassidy, J.P., Smedegaard, B.H., Agger, E.M., and Andersen, P. (2000). Control of latent Mycobacterium tuberculosis infection is dependent on CD8 T cells. Eur. J. Immunol. *30*, 3689–3698.

Verver, S., Warren, R.M., Beyers, N., Richardson, M., van der Spuy, G.D., Borgdorff, M.W., Enarson, D.A., Behr, M.A., and van Helden, P.D. (2005). Rate of reinfection tuberculosis after successful treatment is higher than rate of new tuberculosis. Am. J. Respir. Crit. Care Med. *171*, 1430–1435.

Wang, X., Cao, Z., Jiang, J., Li, Y., Dong, M., Ostrowski, M., and Cheng, X. (2011). Elevated expression of Tim-3 on CD8 T cells correlates with disease severity of pulmonary tuberculosis. J. Infect. *62*, 292–300.

Wheaton, W.W., Weinberg, S.E., Hamanaka, R.B., Soberanes, S., Sullivan, L.B., Anso, E., Glasauer, A., Dufour, E., Mutlu, G.M., Budigner, G.S., and Chandel, N.S. (2014). Metformin inhibits mitochondrial complex I of cancer cells to reduce tumorigenesis. eLife *3*, e02242.

Wherry, E.J., and Kurachi, M. (2015). Molecular and cellular insights into T cell exhaustion. Nat. Rev. Immunol. *15*, 486–499.

Wherry, E.J., Ha, S.J., Kaech, S.M., Haining, W.N., Sarkar, S., Kalia, V., Subramaniam, S., Blattman, J.N., Barber, D.L., and Ahmed, R. (2007). Molecular signature of CD8+ T cell exhaustion during chronic viral infection. Immunity *27*, 670–684.

Willems, P.H., Rossignol, R., Dieteren, C.E., Murphy, M.P., and Koopman, W.J. (2015). Redox Homeostasis and Mitochondrial Dynamics. Cell Metab. *22*, 207–218.

Zumla, A., Rao, M., Wallis, R.S., Kaufmann, S.H., Rustomjee, R., Mwaba, P., Vilaplana, C., Yeboah-Manu, D., Chakaya, J., Ippolito, G., et al.; Host-Directed Therapies Network consortium (2016). Host-directed therapies for infectious diseases: current status, recent progress, and future prospects. Lancet Infect. Dis. *16*, e47–e63.

STAR★METHODS

KEY RESOURCES TABLE

REAGENT or RESOURCE	SOURCE	IDENTIFIER
Antibodies		
Anti-CD16/CD32	Biolegend	Cat# 101302
Anti-CD3/CD28 Dynabeads	Life Technologies	Cat# 114.53D
anti-CD45 (30-F11) AF700 Rat Anti-Mouse	BD Biosciences	Cat# 560510
anti-CD3 (17A2) PE-Cy7 Rat Anti-Mouse CD3 Molecular Complex	BD Biosciences	Cat# 560591
anti-CD4 (RM4-5) BV605 Rat Anti-Mouse	BD Biosciences	Cat# 563151
anti-CD8a (53-6.7) V500	BD Biosciences	Cat# 560776
anti-IFNγ (XMG1.2) BV786	BD Biosciences	Cat# 563773
anti-CD44 (IM7) BV650	Biolegend	Cat# 103049
anti-PD-1 (29F.1.A12) PE Anti-Mouse CD279	Biolegend	Cat# 135205
anti-KLRG-1 (2F1/KLRG1) APC	Biolegend	Cat# 138411
anti-CTLA4 (UC10-4B9) PerCP/Cy5.5	Biolegend	Cat# 106315
anti-TNFα (MP6-XT22) BV11 Anti-mouse	Biolegend	Cat# 506349
anti-IL-10 (JES5-16E3) PerCP/ Cy5.5	Biolegend	Cat# 505028
anti-IL-2 (JES6-5H4) FITC	Biolegend	Cat# 503805
anti-IL-2 (JES6-5H4) PE-Dazzle 594	Biolegend	Cat# 503840
Bacterial and Virus Strains		
Mycobacterium tuberculosis (*Mtb*) strain H37Rv	ATCC	Cat# 27294
Mycobacterium bovis, Danish BCG vaccine strain 1331 (BCG)	Stratens Serum Institut	N/A
Chemicals, Peptides, and Recombinant Proteins		
Metformin hydrochloride (Glucophage 500mg film-coated tablets)	Merck	Reg No: 41/21.2/0639
Middlebrook 7H11 Agar	BD Biosciences	Cat# 283810
Middlebrook OADC	BD Biosciences	Cat# 212240
Middlebrook 7H9 Broth	BD Biosciences	Cat# 271310
Collagenase D	Roche	Cat# 11088866001
DNase	Roche	Cat# 10104159001
ACK Lysis Buffer	Lonza	Cat# 10-548E
Cell-tak	Corning	Cat# 354241
RPMI1640	Lonza	Cat# BE12-167F
GlutaMax	GIBCO	Cat# 35050-038
Oligomycin (from Streptomyces diastatochromogenes)	Sigma	Cat# O4876
carbonilcyanide p-triflouromethoxyphenylhydrazone (FCCP)	Sigma	Cat# C2920
Antimycin A	Sigma	Cat# A8674
Rotenone	Sigma	Cat# R8875
Phorbol 12-myristate 13-acetate	Sigma	Cat# P8139
Ionomycin Calcium Salt	Sigma	Cat# 10634
Brefeldin A	Sigma	Cat# B7651
K^b TB10.4-TET$_{4-11}$ (IMYNPAM) MHC class I tetramer	NIH Tetramer Core Facility	N/A

(*Continued on next page*)

Continued

REAGENT or RESOURCE	SOURCE	IDENTIFIER
Critical Commercial Assays		
RNeasy Micro kit	QIAGEN	Cat# 74004
iMAG CD8 T Lymphocyte Enrichment kit	BD	Cat# 558471
Foxp3 Intracellular Staining Kit	eBioscience	Cat# 00-5523-00
LIVE/DEAD Fixable Dead Cell Stain 633/635nm excitation	Life Technologies	Cat# L10119
MitoTracker Green (MTG)	Life Technologies	Cat# M7514
Mitotracker Deep Red (MTDR)	Life Technologies	Cat# M22426
MitoSox Red	Life Technologies	Cat# M36008
2-[N-(7-nitrobenz-2-oxa-1,3-diazol-4-yl) amino]-2-deoxy-D-glucose (2-NBDG)	Life Technologies	Cat# N13195
Seahorse XF Cell Mito Stress Test Kit	Agilent Technologies	Cat# 103015-100
Seahorse XF Glycolysis Stress Test Kit	Agilent Technologies	Cat# 103020-100
Deposited Data		
Raw and analyzed data	This paper	GEO: GSE117330
Mouse reference genome Gencode Release M11, GRCm38 p4	Gencode	https://www.gencodegenes.org/
Experimental Models: Organisms/Strains		
Mouse: C57BL/6J	Bred in-house (AHRI)	N/A
Software and Algorithms		
Wave desktop software, Version 2.6	Agilent	https://www.agilent.com/en/products/cell-analysis/cell-analysis-software/data-analysis/wave-desktop-2-6
Bcl2fastq version 2.18.0.12	Illumina	https://www.illumina.com/index-d.html
STAR version 2.5.3a	Dobin, et al. 2013	https://github.com/alexdobin/STAR
HTSeq-count version 0.9.1	Anders, et al. 2015	https://htseq.readthedocs.io/en/release_0.11.1/index.html#
DESeq2 version 1.24.0	Love, et al. 2014	https://bioconductor.org/packages/release/bioc/html/DESeq2.html
Ingenuity Pathway Analysis	QIAGEN	https://www.qiagenbioinformatics.com/
Skyline V3.7	MacLean, et al. 2010	https://skyline.ms/project/home/software/Skyline/begin.view
Analyst software package, Version 1.6.2	AB/MDS Sciex	https://sciex.com/products/software/analyst-software

LEAD CONTACT AND MATERIALS AVAILABILITY

Further information and requests for resources and reagents should be directed to and will be fulfilled by the Lead Contact, Dr. Adrie JC Steyn (asteyn@uab.edu). This study did not generate unique reagents.

EXPERIMENTAL MODEL AND SUBJECT DETAILS

Mice

Six- to eight-week old female C57BL/6J mice (bred in-house at the Africa Health Research Institute) were used for all *in vivo* experiments. Littermates were randomly assigned to experimental groups. All mice were housed 5 mice per cage and were given environmental enrichment. Food and water was provided *ad libitum*. All procedures were approved by the Animal Ethics Sub-Committee at the University of KwaZulu-Natal (reference number AREC/003/016PD). Mice were cared for in accordance with South African national guidelines.

Bacteria

Mycobacterium tuberculosis (Mtb), strain H37Rv (ATCC 27294) and *Mycobacterium bovis* BCG (Danish 1331 strain, Statens Serum Institut) were cultured in 7H9 medium (BD) supplemented with 10% OADC (BD). Bacteria were cultured to an O.D. of 1.0 and diluted 1/100 in 7H9/OADC medium prior to aerosol infection.

METHOD DETAILS

Study design

According to a study by Gideon et al. (2015) only a small fraction (8%) of T cells in TB granulomas produce cytokines when re-stimulated with extracts from *Mtb*-infected lungs, *Mtb*-specific peptides or *Mtb*-infected dendritic cells, suggesting that many T cells recruited to these lesions cannot produce cytokines because (1) cytokine production is inhibited, or (2) the majority of T cells recruited are not specific for mycobacterial antigens. Based on this work and others, in this study we chose to view the *Mtb*-infected lung holistically. In most instances, we compare the immunometabolic signatures represented by the total $CD8^+$ T cell populations from uninfected, BCG and *Mtb*-infected mice. We believe there is strong justification for this approach because of the complex, multicellular architecture of TB lesions, especially considering the broad effects imposed on these cells during chemotherapy. To provide additional context, we compare these readouts with a subset of *Mtb*-specific $CD8^+$ T cells ($TB10.4_{4-11}$-specific), acknowledging that the immunometabolic characteristics we observe may not be generalizable to all *Mtb*-specific $CD8^+$ T cells. We anticipate this study design will provide the basis for further research, particularly as it pertains to developing innovative approaches to test candidate host-directed therapies or assess vaccine efficacy.

We chose to include BCG in this study because of its relevance as the most widely used vaccine strain globally, its extensive use in laboratory research settings, and its mild clinical presentation, for comparative purposes with virulent *Mtb*.

Bacterial infections and drug treatment

Six- to eight-week old C57BL/6J mice (bred in-house) were aerosol-infected with ~100 CFU of virulent *M. tuberculosis*, strain H37Rv (ATCC 27294) or *M. bovis* BCG (Danish 1331, Statens Serum Institut) using a Glas-Col Aerosol Exposure System. Mice were euthanized on day 21, day 35 and 12 weeks post-infection by cervical dislocation under anesthesia (isoflurane), and lungs and spleen were aseptically removed. For the drug experiments, 7 days post-infection, mice were treated with metformin hydrochloride by oral gavage (500 mg/kg/day) 5 days per week for 4 weeks.

Bacterial enumeration and T cell isolation

Individual mouse lungs were homogenized in 10 mL sterile PBS/0.02% Tween 80 using a gentleMACS Dissociator (Miltenyi). Bacteria were enumerated by plating 10-fold serial dilutions onto 7H11 agar (BD) supplemented with 10% OADC (BD) and PACT (Sigma). Colonies were counted 3-4 weeks later. For functional assays, lungs were perfused with PBS to eliminate contaminating blood cells, homogenized in 5 mL 0.5mg/mL Collagenase D with 20 μg/mL DNase (Roche), incubated for 30 min at 37°C, then passed through a 70 μM cell strainer (BD). Red blood cells were lysed with ACK lysis buffer (GIBCO) and remaining cells were prepared for downstream analyses. $CD8^+$ T cells were purified from white blood cell suspensions by negative magnetic bead selection using the Mouse CD8 T Lymphocyte Enrichment kit (iMAG, BD).

RNA preparation, sequencing, and analysis

RNA was extracted from 1.2×10^6 $CD8^+$ T cells from UI and infected (BCG or *Mtb*) mouse lungs using the RNeasy Micro kit (QIAGEN). RN/RQ values were measured using the Agilent 2100 Bioanalyzer. Samples for RNASeq were isolated from 5 mice per group. Three of the highest quality samples (based on RN/RQ values) were submitted for sequencing at the University of Alabama Heflin Genomics Core Facility.

mRNA sequencing was performed on the Illumina NextSeq500 as described by the manufacturer (Illumina). Library preparation was performed using the SureSelect Strand Specific mRNA library kit as per the manufacturer's instructions (Agilent). Library construction began with two rounds of polyA selection using oligo dT-containing magnetic beads. The resulting mRNA was randomly fragmented with cations and heat, followed by first strand synthesis using random primers with inclusion of Actinomycin D (2.4ng/μL). Second strand cDNA production was performed using standard techniques and the ends of the resulting cDNA were blunted, A-tailed and adaptors ligated for indexing to allow for multiplexing during sequencing. cDNA libraries were quantitated using qPCR (Roche, LightCycler 480) with the Kapa Biosystems kit for Illumina library quantitation (Kapa Biosystems) prior to cluster generation. Cluster generation was performed according to manufacturer's recommendations for onboard clustering (Illumina). Paired-end 75bp sequencing runs were used to allow for better alignment to the reference genome.

STAR (version 2.5.3a) was used to align the raw RNA-Seq fastq reads to the mouse reference genome (GRCm38 p4, Release M11) from Gencode (Dobin et al., 2013). Following alignment, HTSeq-count (version 0.9.1) was used to count the number of reads mapped to each gene (Anders et al., 2015). Normalization and differential expression was then applied to the count files using DESeq2 (Love et al., 2014). Genes considered significant Genes considered significant had a fold-change value $\geq +/-2$. Principal component analysis (PCA) plots were generated using the normalized dataset in DESeq2. Heatmap generation was done in R version 3.4.3 using gplots version 3.0.1 heatmap.2 function. In brief, fold change values for each group were loaded into R as a data matrix. The gplots

heatmap.2 was then used to plot the data matrix using the arguments: scale = ”row,” key = T, keysize = 1.5, col = bluered (75), density.info = ”none,” trace = ”none,” labRow = F. Raw sequence reads were uploaded to NCBI Gene Expression Omnibus (GSE117330).

Gene set enrichment analysis

To generate networks, a dataset containing gene identifiers and corresponding expression values was uploaded into Ingenuity Pathway Analysis (QIAGEN). Each identifier was mapped to its corresponding object in Ingenuity's Knowledge Base. A fold change cutoff of $\pm$ 2 and p value < 0.05 was set to identify molecules whose expression was significantly differentially regulated. These molecules, called Network Eligible molecules, were overlaid onto a global molecular network developed from information contained in Ingenuity's Knowledge Base. Networks of Network Eligible Molecules were then algorithmically generated based on their connectivity. The Functional Analysis identified the biological functions and/or diseases that were most significant to the entire dataset. Molecules from the dataset that met the fold change cutoff of $\pm$ 2 and p value < 0.05, and were associated with biological functions and/or diseases in Ingenuity's Knowledge Base were considered for the analysis. Right-tailed Fisher's exact test was used to calculate a p value determining the probability that each biological function and/or disease assigned to that dataset was due to chance alone.

Metabolic extracellular flux analysis

Seahorse extracellular flux (XF) experiments were performed on total $CD8^+$ T cells from UI and infected mice purified using negative magnetic bead selection as described above. *Mtb*-specific TB10.4-tetramer$^+$ cells were sorted using the FACSAria II (BD Biosciences). Total $CD8^+$ T cells were sorted alongside tetramer$^+$ cells to serve as sorted controls. CMST parameters were measured in XF media (non-buffered RPMI 1640 medium, containing 25 mM glucose, 2 mM L-glutamine, and 1mM sodium pyruvate). Cells (250,000 live cells/well) were adhered to the plate using Cell-Tak (Corning) as previously described (Kramer et al., 2014). Briefly, 10 μL of Cell-Tak reagent (135 mL Cell-Tak, 270 mL dH2O, 810 mL of 0.1 N sodium bicarbonate (pH 8.0), followed by adjusting the pH to 7.2–7.8 with 1 N NaOH) was added to each well of the XF plate, incubated for 30 min at 37°C and then washed off using three rinses with dPBS. Cells were spun down onto the XF plate at 1500 rpm for 5 min with the break off, and the plate with flipped around and the same spin was done with the plate in the opposite orientation to create a uniform monolayer of cells across the bottom of the well. Cells were equilibrated for 1h at 37°C, and assayed for OCR and ECAR under basal conditions, and in response to 1.25 μM oligomycin, 1.5 μM FCCP, 200 μM etomoxir, 25 mM 2-deoxyglucose (2-DG), 100 nM rotenone/1 μM antimycin A (all Sigma). Activation bioenergetics were measured by incubating purified $CD8^+$ T cells with anti-CD3/anti-CD28 Dynabeads (Life Technologies) for 1h at 37°C prior to initiating the run. In the glycolysis stress test (GST) assay, cells were equilibrated in glucose-free media for 1h prior to initiating the run and glucose was injected (10 μM) at the beginning of the run to measure the cells' dependency on glycolysis. Cells were assayed on an XF-96 Extracellular Flux Analyzer (Agilent). Measurements were normalized to total μg protein in each well using a Bradford assay (BioRad).

Briefly, in the CMST, injection of oligomycin, an inhibitor of ATP synthase, provides an estimate of mitochondria-derived ATP production, injection of FCCP, a mitochondrial uncoupler, pushes the cell to its maximal respiratory capacity (MRC), and injection of antimycin A and rotenone shuts down the electron transport chain, revealing non-mitochondrial oxygen consumption. The CMST can also quantify a cell's spare respiratory capacity (SRC), which is a measurement of the cell's ability to increase respiration when the energy demand exceeds supply - under conditions of stress or increased workload.

Bioenergetic phenograms were generated by plotting OCR as a function of ECAR after FCCP addition to illustrate how mitochondrial stress impacts the energy phenotype of the cell population in question. The energy phenotype of cells can be described as more aerobic, energetic, glycolytic or quiescent.

MHC tetramers, antibodies, and flow cytometry

For mitochondrial assays, purified cells were incubated with 50 nM MitoTracker Green (MTG) and 12.5 nM Mitotracker Deep Red (MTDR) in RPMI 1640 (GIBCO) containing 10% FBS, 2 mM L-glutamine for 30 min at 37°C prior to staining. Mitochondrial superoxides were assessed using 5 μM MitoSox Red incubated for 10 min at 37°C in HBSS. Glucose uptake was assayed by incubating cells with 100 μM 2-[N-(7-nitrobenz-2-oxa-1,3-diazol-4-yl) amino]-2-deoxy-D-glucose (2-NBDG) at 37°C in glucose-free RPMI 1640 (GIBCO) containing 10% FBS, 2 mM L-glutamine for 30 min prior to FACS staining. MTG, MTDR, MitoSox and 2-NBDG were obtained from Life Technologies.

Intracellular cytokine production in purified cells was assessed by incubating $1x10^6$ cells with 50 ng/mL phorbol 12-myristate 13-acetate (PMA) and 1 μg/mL ionomycin for 4 h. Brefeldin A (10 μg/mL) was added 1 h prior to surface staining. All reagents were obtained from Sigma. With the likelihood that only a small percentage of $CD8^+$ T cells produce cytokines in response to *Mtb* antigens (Gideon et al., 2015), we chose the non-specific re-stimulation method PMA/ionomycin because it could be broadly applied to all three groups of T cells (UI, *Mtb*-infected, BCG-infected) for comparison purposes.

For flow cytometry analyses, purified cells were blocked with rat anti-mouse CD16/CD32 (Biolegend) and stained with anti-CD45 (30-F11), anti-CD3 (17A2), anti-CD4 (RM4-5), anti-CD8a (53-6.7), anti-IFN-γ (XMG1.2)(BD Biosciences), anti-CD44 (IM7), anti-PD-1 (29F.1.A12) anti-KLRG-1 (2F1/KLRG1), anti-CTLA4 (UC10-4B9) anti-TNFα (MP6-XT22), anti-IL-10 (JES5-16E3, anti-IL-2 (JES6-5H4) (Biolegend). K^b TB10.4-TET_{4-11} (IMYNPAM) MHC class I tetramer was obtained from the NIH Tetramer Core Facility (Emory University, Atlanta, GA). Cells were stained with tetramer (1:200) and/or extracellular antibodies at 4°C for 30 min. If required, cells were fixed

and permeabilized using the Foxp3 Intracellular Staining Kit (eBioscience). Prior to antibody staining, cells were stained with the LIVE/DEAD Fixable Dead Cell Stain (Life Technologies) to exclude dead cells from the analysis. Flow cytometry was performed using an LSRII Fortessa or FACSAriaII (BD Biosciences) and data were analyzed with FlowJo V10.1 software (TreeStar).

Importantly, all TB10.4-TET$^+$ cells described in this study refer to antigen-specific T cells from *Mtb*-infected mice, not BCG; although there is a TB10.4 homolog in BCG, we were unable to detect TB10.4-TET$^+$ cells in BCG-infected mice.

Carbon Tracing, Metabolite Extraction and Mass Spectrometry Analysis

Carbon tracing experiments were performed on total CD8$^+$ T cells purified as described above. 1x10^6 cells were cultured at 37°C with 5% CO_2 for 1 h in 1 mL glucose-free RPMI 1640 (GIBCO) containing 10% FBS, 2 mM L-glutamine and 25 mM $^{13}C_6$-glucose (Sigma). Metabolites were extracted in 700 μL 50:50 H_2O:MeOH containing 51.2 mg/mL para-nitrophenyl-phosphate (pNPP) standard (Sigma). Samples were stored at −80°C overnight and spun through a 0.22 μM filter (Corning), dried down and reconstituted in 100 μL ddH_2O. Metabolites were separated on a Biorad Aminex HPX-87 column (300 × 7.8mm), using an aqueous 0.1% formic acid isocratic mobile phase, connected to a Dionex Ultimate 3000 UPLC. Total negative ion chromatograms (50 – 750 m/z scan range) were collected using a Thermo Scientific Q Exactive mass spectrometer. The total ion chromatograms of all relevant metabolites, and their respective isotopologues, were visualized and analyzed using Skyline V3.7 (MacLean et al., 2010).

AXP levels were quantified using methods described previously with minor modifications (Lamprecht et al., 2016). Metabolite extracts were prepared from CD8$^+$ T cells as described above, purified in RPMI 1640 media containing unlabeled glucose. Extracts were run on the Agilent 1200 series binary HPLC system (Agilent) coupled to an Applied Biosystems/MDS Sciex QTRAPTM 5500 linear accelerator trap mass spectrometer (AB/MDS Sciex) fitted with an ESI source. The mass spectrometer was operated in negative ion mode and samples were analyzed using multi reaction monitoring (MRM). Data were acquired and analyzed using the Analyst (Version 1.6.2) software package (AB/MDS Sciex). The ATP/ADP/AMP precursor ion/product ions monitored were the following respectively: 505.9 m/z with 159 m/z and 78.8 m/z, 425.9 with 133.9 m/z and 78.9 m/z, and 345.9 m/z with 96.8 m/z and 78.9 m/z. AXP levels were quantified using a method of standard addition and normalized to total ug protein as measured by Bradford assay (BioRad).

QUANTIFICATION AND STATISTICAL ANALYSIS

Differences between control and experimental groups were compared using a one-way analysis of variance (ANOVA) with multiple comparisons to calculate statistical significance (GraphPad Prism software, version 7.0). In instances where only two groups were compared, an unpaired Student's t test was used (GraphPad Prism). All animal experiments were performed with five mice per experimental group, unless additional mice were required (up to 10 mice/group) for experiments requiring larger cell numbers or for the analysis of cell populations present in lower frequencies (e.g., TB10.4-TET$^+$ cells). Samples were pooled for subsequent XF96 (CMST and GST), metabolomic, intracellular, and extracellular flow cytometry and mitochondrial fitness analyses to ensure all experimental tests could be performed in parallel in a single animal experiment, under BSL3 conditions. No pooling was done for CFU experiments or transcriptomic analyses. Further information with regard to specific experiments, including the number of experimental replicates, can be found in the figure legends.

DATA AND CODE AVAILABILITY

The accession number for the raw RNA-sequencing data reported in this paper is GEO accession number GEO:(GSE117330). All data needed to evaluate the conclusions in the paper are present in the paper and/or the Supplementary Materials except for the transcriptomic data. Raw sequence reads were uploaded to NCBI Gene Expression Omnibus (GSE117330). Additional data related to this paper may be requested from the authors.

Cell Reports, Volume *29*

Supplemental Information

Compromised Metabolic Reprogramming Is an Early Indicator of CD8$^+$ T Cell Dysfunction during Chronic *Mycobacterium tuberculosis* Infection

Shannon L. Russell, Dirk A. Lamprecht, Tawanda Mandizvo, Terrence T. Jones, Vanessa Naidoo, Kelvin W. Addicott, Chivonne Moodley, Bongani Ngcobo, David K. Crossman, Gordon Wells, and Adrie J.C. Steyn

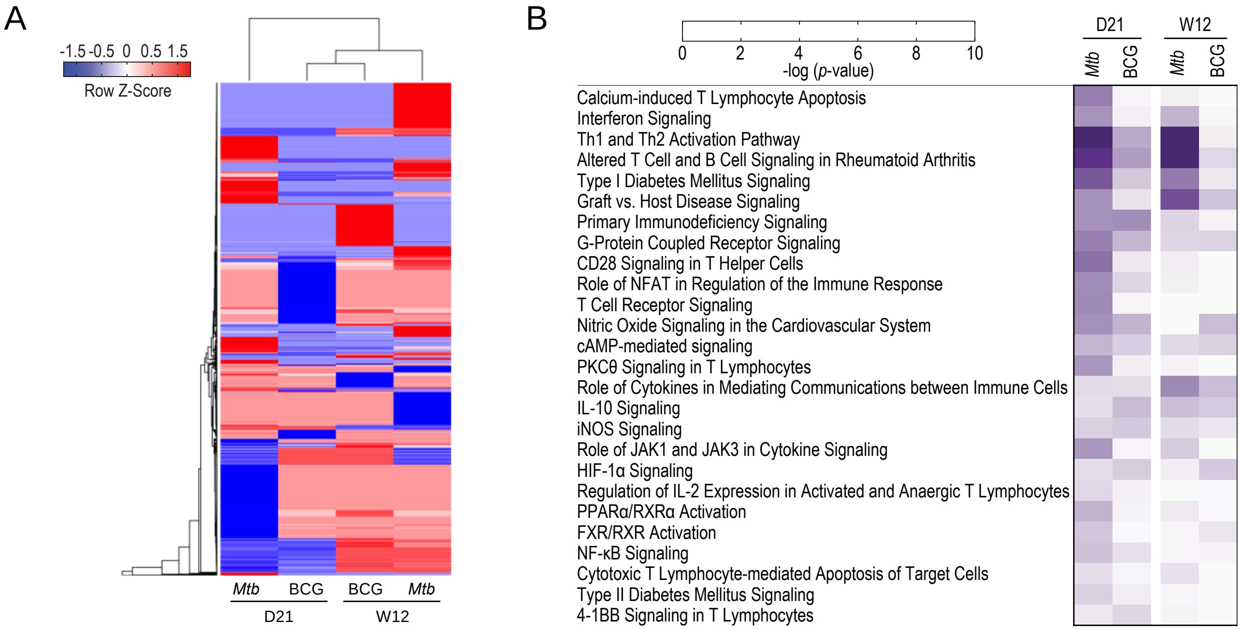

Figure S1. Variation in global gene expression patterns and enriched pathway analysis in lung CD8$^+$ T cells during *Mtb* or BCG infection. (Related to Figure 1.) RNA was extracted from purified CD8$^+$ T cells from the lungs of *Mtb* or BCG-infected mice at D21 and W12, sequenced and analyzed for differences in global gene expression patterns. **(A)** Global differential gene expression patterns of CD8$^+$ T cells from *Mtb* and BCG-infected animals at D21 and W12 (relative to UI). Clustering indicates the relatedness between samples as defined by heatmap.2 in R. **(B)** The top canonical pathways are shown based on enrichment score (Fisher's exact test *P*-value), defined by QIAGEN's Ingenuity® Pathway Analysis software. All comparisons are relative to expression levels of CD8$^+$ T cells purified from the lungs of UI mice.

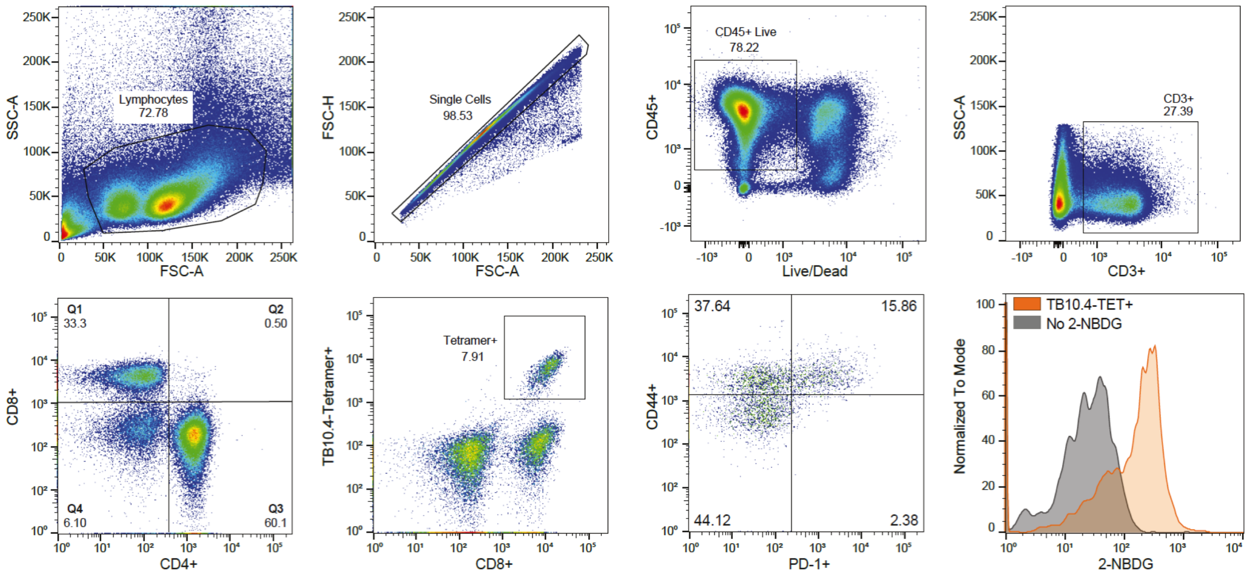

Figure S2. Gating strategy for flow cytometry analysis of CD8$^+$ T cells. (Related to Figure 2.)

Purified single cell suspensions prepared from the lungs of uninfected or infected mice were stained with fluorochrome-conjugated antibodies and analyzed on the BD Fortessa or FACS Aria II. Using FlowJo software (TreeStar, V10.1), total lymphocytes and singlets were gated using FSC and SSC parameters, followed by live, CD45$^+$ cells. Cytotoxic T lymphocytes were identified by gating on the CD3$^+$ population, followed by those that stained CD8$^+$CD4$^-$. *Mtb*-specific CD8$^+$ T cells were identified as those that stained TB10.4 tetramer$^+$ within the CD3$^+$CD8$^+$CD4$^-$ subset. Cells were further gated on CD44$^+$ expression to identify T effectors, and subsequently PD-1 or CTLA-4 (not shown) to examine co-inhibitory receptor expression. The cell population of interest was examined for 2-NBDG uptake (measured by mean fluorescence intensity, or MFI) if glucose uptake was assayed; this was always normalized to the MFI of unstained cells.

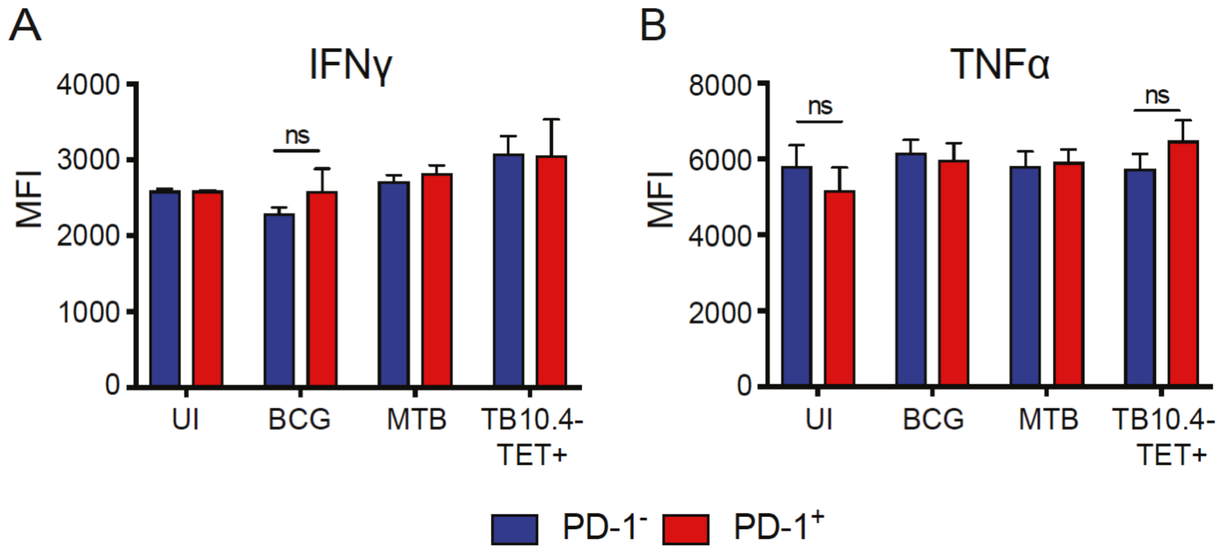

Figure S3. Cytokine production in $CD8^+$ T cells expressing PD-1 receptor. (Related to Figure 2.)

Purified single cell suspensions prepared from the lungs of uninfected or infected mice were stimulated with PMA/ionomycin for 4 hours and then stained with fluorochrome-conjugated antibodies. $CD8^+$ T cells were gated based on their expression of PD-1 receptor, and intracellular cytokine production was measured based on the mean fluorescence intensity of the antibody used to detect the cytokine of interest. Data shown is from D35 post-infection, although reflects similar trends at D21 and W12. Data are representative of three independent experiments (5 mice per experimental group). ns, not significant, by unpaired Student's t-test. Error bars are mean ± SD.

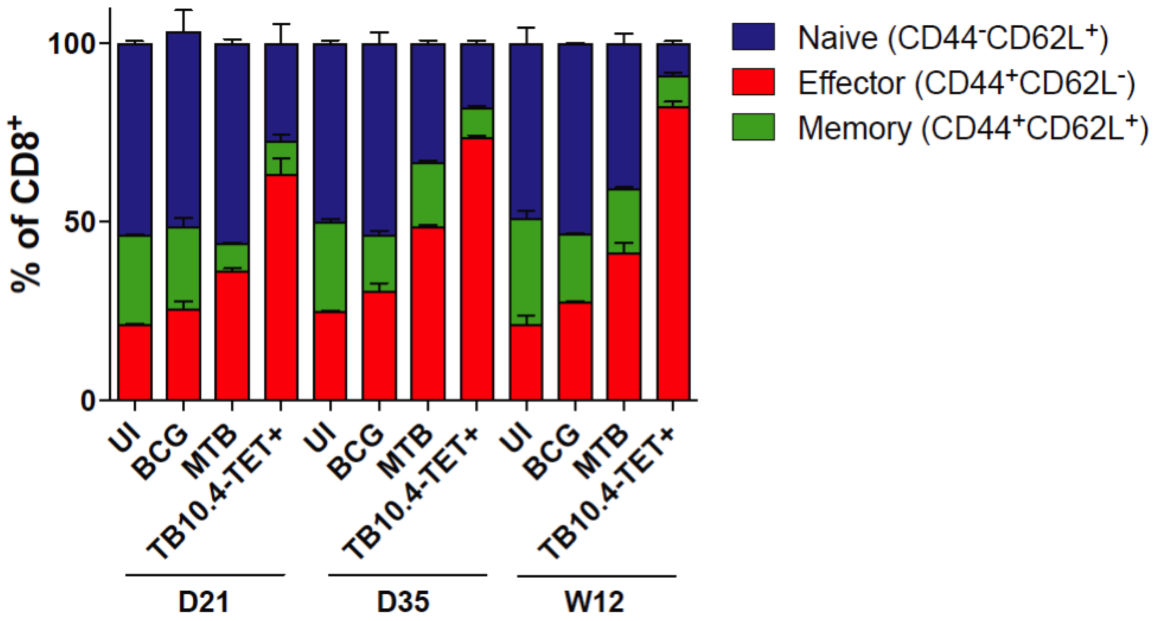

Figure S4. Naïve, effector and memory CD8^{+} T cell populations over time in UI, BCG and *Mtb*-infected mice. (Related to Figure 3.) Expression of CD44 and CD62L were used to estimate the number of naïve (CD44^{-}CD62L^{+}), effector (CD44^{+}CD62L^{-}) and memory (CD44^{+}CD62L^{+}) CD8^{+} T cells present in purified single cell suspensions prepared from the lungs of uninfected or infected mice on D21, D35 and W12 post-infection using unfixed flow cytometry. Data are representative of two independent experiments (5 mice per experimental group). Error bars are mean ± SD.

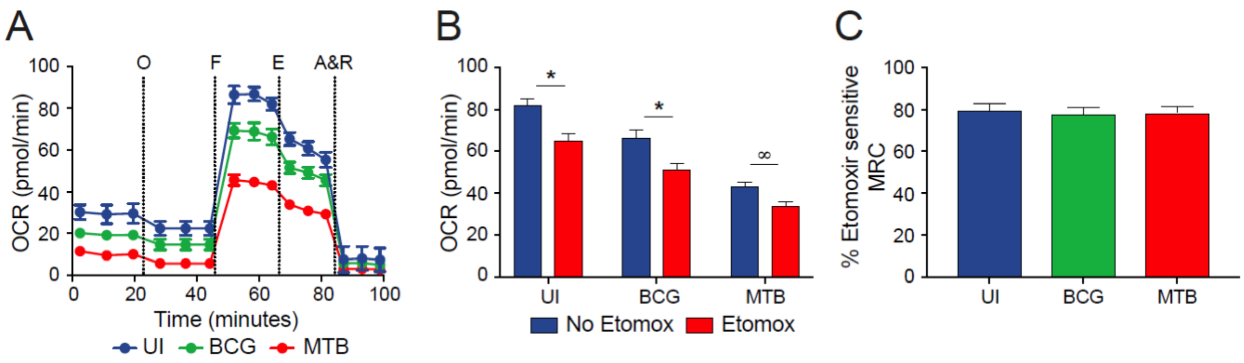

Figure S5. Fatty acid oxidation contributes equally to OCR in CD8$^+$ T cells, regardless of infection status. (Related to Figure 3.) Extracellular flux (XF) analysis was performed on purified lung CD8$^+$ T cells during chronic *Mtb* or BCG infection. **(A)** OCR measured during a cell mito stress test (CMST), performed with an extra infection of the Cpt1a inhibitor, etomoxir (E) after uncoupling with FCCP. **(B)** OCR before and after etomoxir injection (inhibits fatty acid oxidation, FAO) and **(C)** % etomoxir-sensitive OCR (OCR not due to FAO). Data shown is from D35 post-infection, although reflects similar trends at D21 and W12. UI, uninfected; OCR, oxygen consumption rate; ECAR, extracellular acidification rate; O, oligomycin; F, FCCP or carbonyl cyanide p-trifluoromethoxyphenylhydrazone; E, etomoxir; A&R, antimycin A and rotenone. Data are representative of two independent experiments (5 mice per experimental group). ∞ $p \leq 0.005$; * $p \leq 0.001$, by unpaired Student's t-test or one-way ANOVA. Error bars are mean ± SD.

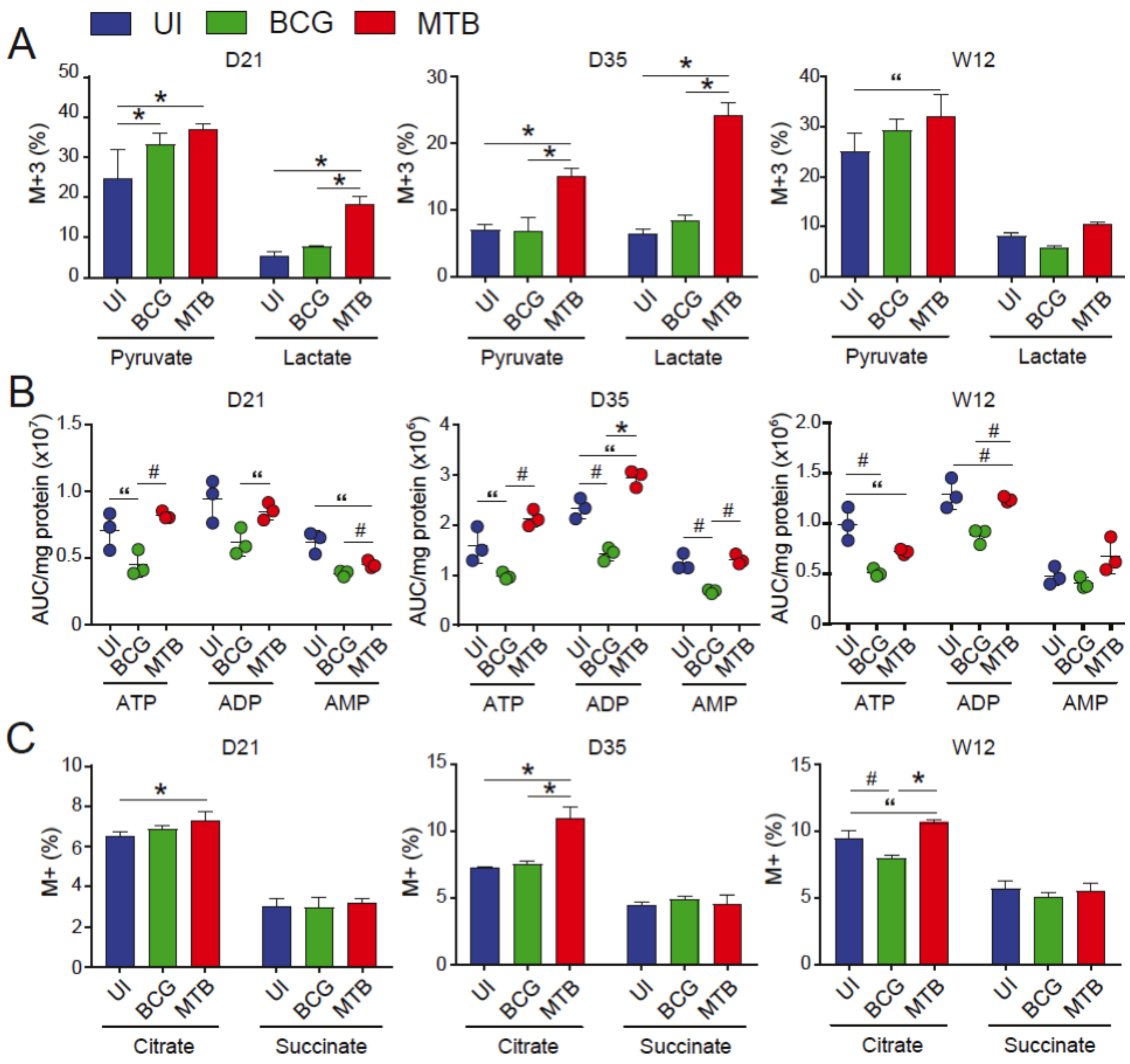

Figure S6. *Mtb* infection increases metabolic flux through glycolysis to generate ATP via substrate-level phosphorylation. (Related to Figure 3.) Metabolite pools were generated in CD8$^+$ T cells purified from UI, *Mtb* or BCG-infected animals at D21, D35 or W12 post-infection following incubation for 1h at 37 °C in ^{13}C-glucose-containing media. **(A)** Pyruvate and lactate M+3 isotopologues, **(B)** absolute ATP, ADP and AMP levels and **(C)** citrate and succinate combined M+ isotopologues detected in metabolite extracts, as quantified by LC-MS. UI, uninfected. Stats unless otherwise indicated, are relative to UI. Data are representative of two independent experiments (N = 5 mice per group). " $p \leq 0.05$; # $p \leq 0.01$; * $p \leq 0.001$, by one-way ANOVA. Error bars are mean ± SD.
